# A History of Thermodynamics: The Missing Manual

**DOI:** 10.3390/e22010077

**Published:** 2020-01-07

**Authors:** Wayne M. Saslow

**Affiliations:** Department of Physics, Texas A&M University, College Station, TX 77843-4242, USA; wsaslow@tamu.edu

**Keywords:** thermodynamics, energy, heat, temperature, entropy

## Abstract

We present a history of thermodynamics. Part 1 discusses definitions, a pre-history of heat and temperature, and steam engine efficiency, which motivated thermodynamics. Part 2 considers in detail three heat conservation-based foundational papers by Carnot, Clapeyron, and Thomson. For a reversible Carnot cycle operating between thermal reservoirs with Celsius temperatures *t* and t+dt, heat *Q* from the hot reservoir, and net work *W*, Clapeyron derived W/Q=dt/C(t), with C(t) material-independent. Thomson used μ=1/C(t) to define an absolute temperature but, unaware that an additional criterion was needed, he first proposed a logarithmic function of the ideal gas temperature $T_{g}$. Part 3, following a discussion of conservation of energy, considers in detail a number of energy conservation-based papers by Clausius and Thomson. As noted by Gibbs, in 1850, Clausius established the first modern form of thermodynamics, followed by Thomson’s 1851 rephrasing of what he called the Second Law. In 1854, Clausius theoretically established for a simple Carnot cycle the condition $Q_{1}/T_{1}+Q_{2}/T_{2}=0$. He generalized it to $\sum_{i}Q_{i}/T_{g,i}=0$, and then $\oint dQ/T_{g}=0$. This both implied a new thermodynamic state function and, with appropriate integration factor 1/T, the thermodynamic temperature. In 1865, Clausius named this new state function the entropy *S*.

## 1. Introduction

An article on the history of thermodynamics can do no worse than begin by citing the first usage of any version of the word *thermodynamics*. This was in an 1849 article by William Thomson referring to “thermo-dynamic engines”, by which he meant devices—in particular, steam engines—that convert heat (thermo) to motion (dynamics) [1]. From 1866, when he was knighted, he was Sir William Thomson; not until 1892, on becoming Baron of Largs, was he known as Lord Kelvin.

Until around 1850 heat and work were considered to be distinct subjects, although by the early 1840s there was growing and consistent quantitative evidence that—in processes where system properties change—work can be converted to heat. Moreover, it was further proposed that some combination of the two was conserved.

Driving the unification of heat and work was the theoretical study of steam engine efficiency, with initial crucial steps made by the engineer Sadi Carnot and then by the engineer Clapeyron, using the “heat function” of Laplace. Only after the conceptual unification of heat and work did physicists bring energy-based thermodynamics to fruition.

Because he saw no evidence that heat could be converted *completely* to work with no other changes to the working system, Thomson initially resisted acceptance of the equivalence of heat and work, and that together they are conserved. Instead he expanded on Clapeyron’s theory of reversible heat engine efficiency based on heat conservation.

Clausius, on the other hand, accepting conservation of energy and building on Carnot, Clapeyron, and Thomson, in 1850 developed the first modern thermodynamic theory (Rather than ‘thermodynamic’, he referred to the ‘moving force of heat’ [2].) He thereby introduced a law based on—all other things constant—heat not flowing from cold to hot [2]. Thomson in 1851, now accepting energy conservation, introduced the noun “thermo-dynamics” and structured what became thermodynamics with two laws, the first being energy conservation [3]. He also introduced a “second” law equivalent to Clausius’s no-cold-to-hot law, based on—all other things constant—heat not completely converting to work. In these works, both Clausius and Thomson used the Celcius temperature scale *t*.

In what follows, where ‘temperature’ is omnipresent, I will employ the usage *ideal gas* temperature scale. Accurately satisfied by dilute gases, in Kelvin units it is given by

(1) $$pv=N\;RT_{g},$$

where *p* is pressure, *v* is the volume, *N* is the number of moles (in the present work, following usage from around 1850, often implicitly taken to be unity), and *R* is the gas constant. $T_{g}$ is approximately related to the Celsius scale *t* by

(2) $$T_{g}=273+t.$$

The issue of temperature scale is a difficult one. Although laboratories currently employ the ideal gas (‘Kelvin’) scale, Thomson first proposed a temperature scale that is, effectively, a logarithmic function of $T_{g}$. His second try was $T_{g}$. Both choices were arbitrary. However, although Thomson provided a consistent driving force to develop thermodynamics, in 1850 Clausius actually developed thermodynamics [4].

Further, in 1854, Clausius brought thermodynamics to a more mature form by establishing the uniqueness of a quantity equivalent to what in 1865 he called the entropy *S*. That is, he showed that, on using the ideal gas temperature scale $T_{g}$ and measuring the heat flow dQ into the system, for any closed path in thermodynamic space $\oint dQ/T_{g}=0$. Thus Clausius established that the entropy $S=\int dQ/T_{g}+S_{0}$ is uniquely defined up to a constant of integration (and therefore is a state function).

In the same stroke Clausius also made possible the thermodynamically well-defined temperature ${T\equiv \partial U/\partial S|}_{V}$, where *V* is the system volume, which is unique (up to an arbitrary temperature-scale factor), and satisfies $T=T_{g}$ when the ideal gas law applies.

**Sources.** The bulk of this article discusses specific fundamental primary sources, but most of the background “pre-history” was obtained from a few important secondary sources. Mendoza’s 1960 *A Sketch for a History of Early Thermodynamics* [5], written by a co-inventor of the helium dilution refrigerator [6], explains for contemporary scientists and engineers how the seemingly naive concept of a conserved heat fluid, *caloric,* could have been accepted for nearly seventy-five years. It also explains how a (now known to be non-physical) heat function, written as Q(p,V), could in Laplace’s hands yield a recognizably modern, if incomplete, theoretical structure. Q(p,V) is not a state function, as assumed by Laplace, since its value depends on its path in (p,v) space. Mendoza’s sketch deliberately restricts its scope, with little on thermometry and heat, and little following Carnot’s classic analysis of 1824.

Cardwell’s 1970 *From Watt to Clausius: The Rise of Thermodynamics in the Early Industrial Age* [7], is far more complete. The remark in his Preface, that thermodynamics typically is taught in a way that “smacks of arbitrary authority”, remains spot-on accurate. This is in part because the equations of thermodynamics, and the physics textbooks that present them, show no trace of their primary motivation—to determine the maximum efficiency of the steam engine, which Carnot established can only be attained for a reversible engine [8]. Like thermodynamics’ contemporary—Lewis Carroll’s Cheshire Cat [9]—for the student all that is left of the story of thermodynamics is the smile (of the equations), and the puzzlement of their origin.

Another useful general reference was Fox’s 1970 *The Caloric Theory of Gases from Lavoisier to Regnault* [10]. This work, with a more chemistry viewpoint, emphasizes calorimetry and the caloric story (proposed by Lavoisier, who concurrently recognized the utility of thinking of heat as produced by mechanical action), and has much pre-Carnot history, culminating with Carnot and then the accurate 1840s thermometric measurements of Regnault.

I also note the valuable 1976 article of Hutchison on “Mayer’s Hypothesis”—any of numerous statements equivalent to assuming that typical gases have constituents that only weakly interact [11]. After discussing Mayer (1842)—the first to publish the principle of energy conservation and to estimate the mechanical equivalent of heat—Hutchison puts the decade-long thermometry studies of Joule and Thomson into perspective.

The above references are at least forty years old. They precede the on-line information revolution, which has made it possible to obtain primary sources without physically entering any library. Except for the introductory material, most of this article relies on primary sources. It is my attempt to provide for the scientific and engineering community “the missing manual” on thermodynamics. It contains both non-technical history and technical discussions of historically important papers. It includes a history of relevant definitions in an attempt to not impose modern usage or ideas in non-chronological order. Hopefully there are no—or at least few—important misconceptions or omissions.

**Analysis.** Besides simply giving the basics of the early history, from Carnot onward I discuss derivations that originally appeared in a number of fundamental papers. In discussing all articles, when available I employ in the equation itself the original numbering; however, in addition I give automatic Latex numbering at the margins. Unfortunately, many of the original articles have no numbered equations at all. In these cases I try to cite equations in such papers by approximate location within a given section (when section numbers exist), or a reprint, as for Carnot [8,12,13] and for Clapeyron [12,14]. Müller [15], Weinberger [16], and Truesdell [17] had helpful bibliographies of original papers. In such cases I use brackets following the equation itself and the Latex numbering; thus the un-numbered 3rd equation in the 12th numbered paragraph (or numbered section) would have an appended [12.3]. I also use brackets to convert partial equations into full equations, so that something like $mc^{2}$ would become $\left[E=\right]mc^{2}$.

**On notation:** different works use volume *V* or *v*, and temperature *T* or *t* (or sometimes even θ or τ). The present work tries to employ consistent usage. However, to avoid being excessively pedantic, I expect the reader to understand the appropriate usage in a number of equations. Typically, specific heats are per unit mass.

**Motivation:** The author’s motivation for the present study began on witnessing, in 1972, following a conference presentation, a disagreement between two distinguished low temperature physicists (John Wheatley—later to receive the 1975 London Prize in Low Temperature Physics—and Robert Richardson—later to receive the 1996 Nobel Prize in Physics). Each employed both noise and magnetic susceptibility thermometry in the millikelvin range, but they disagreed on which was more accurate. In 2018 I realized that, by studying the deviations from the entropy uniqueness condition ΔS=∮dQ/T=0 for *all* paths in *p*-*v* space, one can determine which of two or more temperature scales is “better” [18]. This resolution led me to study how Clausius’s entropy became established in the lore of physics, and the meaningful study of Clausius led to the study of his predecessors, and the present work.

## 2. An Overview

This Overview is lengthy because it is intended to be read profitably independently of the details that follow. It has three parts:

(A) the meaning of temperature;
(B) the pre-history of thermodynamics, with sections on definitions (Section 3), early concepts of heat (Section 4), and then the steam engine (Section 5);
(C) the history of thermodynamics proper, treated with two distinct assumptions:
  (1) conservation of heat (Section 6, Section 7 and Section 8); and
  (2) conservation of energy (Section 9, Section 10, Section 11 and Section 12).

We consider additional topics in Section 13, Section 14, Section 15, Section 16 and Section 17.

### 2.1. Temperature—Material-Independent, Unique, Absolute, Agreement with Ideal Gas

Thermodynamics is more abstract than, say, mechanics, in part because its value is inferred from measurement of a proxy quantity, such as a thermal expansion coefficient or a resistivity.

Moreover, despite Thomson’s high hopes to have found in the Carnot efficiency a *unique* material-independent theoretical basis for temperature, the Carnot efficiency is certainly material-independent, but restricts the temperature scale only to the extent that the lower the temperature $T_{C}$ of the cold reservoir, the higher the thermodynamic efficiency. This permits an infinite number of functional forms for the temperature as a function of the efficiency, restricted only in that the lower the $T_{C}$, the higher the efficiency. As already noted, Thomson proposed two forms for what he the called an absolute temperature (which we take to mean a material-independent temperature). His first (1848) temperature scale yielded the logarithm of his second (1851) temperature scale, given by $T_{g}$ of (1).

However, in Clausius’s hands the Carnot efficiency led to a temperature scale both material-independent and unique. In 1854 he showed that the integration factor 1/T for the heat input dQ, as part of the energy change dU, is equivalent to the ideal gas temperature $T_{g}$. Such equality is not *a priori* obvious, but may not be a fortunate accident, since *T* and $T_{g}$ must have the same units, and there appears to be no intrinsic natural unit for either energy or temperature. This integration factor specification justifies Kelvin’s second scale of $T_{g}$ being the thermodynamic temperature (up to an arbitrary scale factor that permits the Rankine scale).

The reading on a thermal expansion thermometer (such as mercury or alcohol), or a resistivity thermometer, typically is in either Celsius or Fahrenheit. Only because these material-dependent properties are calibrated against a material-independent scale can the readings be accurately interpreted. Except perhaps under extreme conditions, these temperatures are reliable and reproducible. However, they have no special significance except at the negative temperatures that correspond to what in thermodynamics is a special temperature corresponding to the absence of all heat. The ideal gas scale (Kelvin scale) and the Rankine scale are *material-independent, unique, and*—because they agree on that special temperature zero, they are *absolute.*

Concerned about the accuracy of the ideal gas law—studied in detail by Regnault’s laboratory, including small deviations—in 1848 Thomson proposed that for a general heat engine the maximum theoretical efficiency μ can provide a basis for a material-independent temperature scale [19]:

“Is there any principle on which an absolute thermometric scale can be founded? It appears to me that Carnot’s theory of the motive power of heat enables us to give an affirmative answer.”

With *W* the net work and *Q* the heat input, he defined μ via the equivalent of

(3) W=μ(ΔT)Q,

Thomson, without realizing it was not to be his only attempt at a temperature scale, for thermal reservoirs operating between *T* and T−1, so ΔT=1, proposed a temperature scale with μ the same at all temperatures. (His text just before the above quote notes that the experimentally adopted scale is “*an arbitrary series of numbered points of reference sufficiently close for the requirements of practical thermometry.*”) He shortly realized that this scale differed from the ideal gas scale $T_{g}$ so carefully studied by Regnault, and switched to $T_{g}$.

Following a “suggestion” of Joule (no other justification was given), in 1851 Thomson took $\mu ∼T_{g}^{-1}$. He then obtained, for a Carnot cycle with reservoir temperatures $T_{1}$ and $T_{2}$, the equivalent of what I will call the reversible reservoir condition

(4) $$0=\frac{Q_{1}}{T_{1}}+\frac{Q_{2}}{T_{2}}=0.$$

Clausius, in 1854, using the temperature scale $T_{g}$, his already-derived $\mu ∼T_{g}^{-1}$, and valid but unusual physical reasoning, derived the above reversible reservoir condition. He also derived that, for heat dQ, any closed integral in thermodynamic phase space *p*-*v* of $dS=dQ/T_{g}$ is zero. Using the symbol *N*, he wrote:

(5) $$\Delta N=\oint \frac{dQ}{T_{g}}=0.$$

In Clausius’s terminology of 1865, with *S* for *N*, this means the entropy *S* is a state function, so that in the differential dU=dQ−dW for the total energy *U* we have a heat term dQ=TdS. Here, when there is overlap with experiment, $T=T_{g}$. More generally, we consider that the integration factor is defined as 1/T. Because of the integration factor requirement, this temperature *T* is material-independent, unique, and absolute, up to an arbitrary scale factor. Clausius’s argument literally only holds for the zigs and zags associated with isotherms and adiabats, but these zigs and zags may be smoothed out using energy conservation and the fact that *U* and volume *V* are state functions [18].

The modern textbook derivation of (5), which directly calculates the work during a Carnot cycle for the ideal gas, appeared no later than, and perhaps before, 1897 [20]. It could have been obtained (e.g., by Clausius) as early as 1850, since he had derived the adiabatic condition for the ideal gas, needed to calculate the work *W* in a Carnot cycle. Moreover, since Thomson’s derivation of the reversible reservoir condition depended only on the Carnot efficiency, which Clausius had derived, Clausius could have derived the reversible reservoir condition just as had Thomson. Nevertheless, as we note above and discuss in detail in Section 12, in 1854, Clausius was the first to properly derive (5), but by totally different means that the modern derivation. Even more significant is that Clausius alone generalized (4) to sum over a continuous distribution of Carnot cycles; it is this recognition that led to his discovery that $1/T_{g}$ is the integration factor for dQ.

This integration factor requirement would cause other material-independent classes of temperature scales such as the first temperature scale proposed by Thomson, with $\theta =\log\left(T_{g}/273\right)/\left(\log373/273\right)$, to have an awkward integration factor for dS in the energy differential dU. Likewise, if Clausius had used, for example, the material-independent and absolute temperature scale $\theta^{\prime }=T_{g}^{2}$, he would have found the integration factor to be the awkward $1/\theta^{\prime 1/2}=1/T_{g}$.

### 2.2. Outline of Thermodynamics Prehistory

Section 3 addresses the development of modern definitions, with subsections on Force, on Energy and Work, and on Heat and Thermodynamics. This is important because, for example, the modern word “energy” was not used until 1802, in a lecture by Thomas Young at the Royal Institution [21], referring only to $mv^{2}$, the *vis viva* (“living force”) of Leibniz. In other words, “energy” was present as a word, but had a meaning that differed from the more fully developed modern usage. Not even by the time of the 1840s work on energy conservation did physicists use separate words for what we now call force and energy: “kinetic energy”, “potential energy”, “adiabatic” and “entropy” had not yet appeared, and “energy” had to be extended to include potential energy and heat.

Section 4 discusses the evolution of physical thought about fire and heat. Beginning in the early 1700s there were fire-related ideas based on the putative conserved fire-plus-heat fluid *phlogiston* (having mass, and thus *ponderable*), as well as multiple theories involving particles of *fire,* both with and without mass [22]. These were replaced in 1783 by a successor theory that distinguished two conserved quantities: a fire-inducing chemical (oxygen) and the conserved heat-carrying fluid *caloric*. “Specific heat” and “latent heat” had been recently identified and named, but the “calorie” had yet to be defined.

Section 5 discusses the steam engine, which came into use around 1700, and whose efficiency was the motivation for “thermo-dynamics”. The Newcomen engine used cyclic processes, but only with a boiler (hot reservoir). Watt’s engine had the innovation of adding a condenser (“cold” reservoir, but much hotter than room temperature). This additional element made possible Carnot’s analysis [7]. Initially the steam engine, fixed in place, was used primarily to pump water out of mines, and later to supplant water power for textile mills; not until 1805 did Trevethick use a mobile steam engine to power a locomotive [7].

### 2.3. Outline of Thermodynamics Based on Conservation of Heat

The theory of thermodynamics proper initially employed heat conservation but eventually employed energy conservation. It was driven by considerations of maximizing steam engine efficiency. Physics and chemistry texts on thermodynamics typically do not recognize the debt the subject owes to practical considerations, thus giving the impression that thermodynamics was developed solely to provide a consistent macroscopic description of certain properties of materials in equilibrium. However, Clausius’s set of nine memoirs on what we now call thermodynamics acknowledges its origins: *The Mechanical Theory of Heat: With Its Applications to the Steam-engine and to the Physical Properties of Bodies* [2].

Section 6 discusses the academically-trained Carnot’s 1824 contributions to determine the maximum theoretical efficiency of the steam engine. To the ideas of cyclic processes and two thermal reservoirs Carnot added two essential concepts: (1) that for maximum efficiency of conversion of heat to work, no heat transfer should occur unless the working medium and either heat reservoir are at nearly the same temperature, and (2) that one should consider reversible heat engines. He also used the established idea that there is no such thing as a perpetual motion machine. This led Carnot to invent his four-leg process, with two isothermal legs (for the hot and cold reservoirs) and two thermally-insulated (adiabatic) legs for compressive heating or expansive cooling while not in contact with either thermal reservoir. Apparently addressed to a practicing, rather than academic, engineering audience familiar with the steam engine, he presented his work mostly in numerical form, with the exception of one lengthy calculus-based footnote. Despite using heat as a conserved quantity, Carnot’s definition of efficiency of production of work (in modern form e=W/Q) applies to analyses based on energy conservation because he considered that the heat was provided by the hot reservoir, or $Q=Q_{H}$.

Section 7 discusses Clapeyron’s 1834 paper, which put Carnot’s into a more mathematical framework and considered some additional issues. Clapeyron studied a Carnot cycle between two reservoirs at Centigrade temperatures *t* and t+dt, with heat *Q* leaving the hot reservoir and entering to cold reservoir and *W* the net work. He derived that the efficiency e=Q/W=dt/C(t), with C(t) being material-independent. In addition Clapeyron derived an equation equivalent to what we now call the Clausius-Clapeyron equation. For a first order transition this relates the slope of the melting curve to the latent heat and the volume change across the transition. He also related the specific heats at constant volume $c_{v}$ and constant pressure $c_{v}$, and analyzed aspects of the steam engine, recognizing that steam is a complex substance, containing both water droplets and air-water vapor.

Section 8 considers Thomson’s 1848 and 1849 works. The Paris thermometric laboratory of Regnault was host, in 1845–1846, to the young William Thomson [7]. Regnault’s measurements yielded accurate and reproducible values for temperature, but lacked a proper material-independent theoretical basis. Thomson concluded, on reading Clapeyron’s article, that steam engine efficiency could provide that basis, but did not realize at that time that it could only give a non-unique ordering of temperatures. Thomson’s 1848 article, based on Clapeyron’s development of Carnot’s work, invoked the previously-noted efficiency-based definition of temperature and the additional assumption of constant efficiency for constant finite dt. As noted, in the limit as dt→0, this gives a logarithm of $T_{g}$. He then used this to extract a material-independent temperature scale from Regnault’s data. (He shortly abandoned this definition.). Thomson’s 1849 article, written after obtaining a copy of Carnot’s book, re-introduced Carnot’s work to the scientific community.

### 2.4. Outline of Thermodynamics Based on Conservation of Energy

Section 9 discusses the mechanical equivalent of heat and the concept of total energy conservation, topics that mark a watershed in the analysis of steam engine efficiency and the development of thermodynamics. (Kuhn discusses as many as a dozen players on this field, arguing for simultaneous discovery of energy conservation [23]). In 1842 the physician Mayer argued that heat can be converted to work; from the difference between the gas specific heats at constant volume and pressure, and assuming negligible interaction between the gas constituent, he estimated the mechanical equivalent of heat. Somewhat earlier, Joule had begun studying various forms of heat production, starting with electric currents (Joule heating, 1842), and eventually producing heat directly by mechanical work, the famous paddle-wheel experiment; each method gave more or less the same value for the mechanical equivalent of heat. Independently and largely unknown outside of his native Denmark, from 1843 the engineer Colding performed experiments like those of Joule. In 1845 Holtzmann discussed the mechanical equivalent of heat but did not go so far as to conclude that something like energy is conserved. Citing various experiments, especially those of Joule, Helmholtz in 1847 made two distinct contributions: he showed analytically that central forces conserve mechanical energy, and he argued that in the general case, when heat is included, the total energy (‘force’) is conserved. By 1850 Joule had an accurate value for *J* [7].

Section 10 discusses the 1850 work of Clausius, which used conservation of energy and much of the structure of Clapeyron’s and Thomson’s heat-conservation-based 1849 paper, and thereby developed the first modern theory of thermodynamics. He explicitly stated the principle of conservation of energy and added the principle that heat cannot flow from cold to hot if, as for cyclic processes, there are no other changes in the system. He analyzed the Carnot cycle in some detail, obtained the adiabatic condition for an ideal gas, and related the specific heats $c_{v}$ and $c_{p}$. He also considered the steam engine. However, he did not calculate the thermodynamic efficiency of a reversible ideal gas Carnot cycle.

Section 11 considers Thomson’s 1851 works. Citing Clausius, in Part I Thomson finally accepted Joule’s work, and stated what became the first two laws of thermodynamics, the first being energy conservation (citing Mayer and, especially, Joule) and the second being, in effect, that heat cannot be converted directly to work with no other changes (citing Carnot and Clausius). He showed that this is equivalent to Clausius’s statement of the second law. Part II derived the equivalent of the reversible reservoir condition (4). Part III derived the general form for the difference between the two specific heats at constant pressure and constant volume, a staple of modern thermodynamics courses.

Section 12 discusses Clausius’s 1854 article developing an early version of the path-independence of entropy, $\oint dQ/T_{g}=0$. With a complex and unique chain of reasoning, Clausius determined what he called the “equivalence value” (for the transformation of heat, later to lead to entropy). He invoked two different equivalences, one for heat entering or leaving a single reservoir, and one for the same heat leaving a second reservoir and entering a third. He further considered an infinite sum of Carnot cycles, and took the limit, to obtain $\oint dQ/T_{g}=0$. That same year Thomson stated an equivalent relation, based on Joule’s suggestion that *μ*~*T_g_*, for an arbitrary number of coupled Carnot cycles; Thomson did not take the limit to obtain the integral form.

### 2.5. Additional Topics

Section 13 considers the Thomson-Joule work of 1854, on the thermodynamic temperature scale Thomson had already studied in 1851.

Section 14 discusses two priority disputes that appeared in successive published letters between Clausius and Thomson. Thomson argued for Joule’s priority in a “suggestion” that the efficiency $\mu ∼T_{g}$ in an unpublished letter of 1848, as opposed to Clausius derivation of 1850. In my opinion Clausius had the stronger case, but did not argue it as well or as persistently as Thomson and his supporters. In addition, Thomson argued for his friend Joule’s priority in establishing energy conservation and the mechanical equivalent of heat because of Joule’s experiments (of which Mayer performed none); here I would declare a draw.

Section 15 discusses Clausius’s work of 1865 where he developed the name and concept of *entropy* to replace his “equivalence value” of 1854, and stated that on including dissipative processes entropy is a non-decreasing function of time.

Section 16 briefly considers the work of Rankine, which despite numerous deep insights suffers from imprecision and shaky foundations, both mathematically and conceptually. Overall it seems to be held together by comparison to experiment and to the work of Thomson and Clausius, and it does not have the rigor of a deductive theory that begins with well-stated assumptions. In my opinion, Rankine’s work is worthy of further study, because there appears to be much wheat amidst the chaff.

Section 17 discusses the issue of ‘wasted energy’, which gave concern to Thomson, and of entropy production. Because it includes a contribution from particle diffusion, entropy production is a more general property that includes ‘wasted energy’. Entropy production was not properly treated until Onsager’s works of 1931, by which time Thomson’s concern was forgotten.

## 3. On Definitions

It took many years before terms like energy, temperature, heat, work, and adiabat obtained their modern operational and mathematical definitions, no less their modern names. In a hypothetical conversation, modern day scientists and engineers on the one hand, and the founders of thermodynamics on the other hand, might not know what the other group was talking about.

### 3.1. Force

To help the reader better appreciate the issue of definitions, I recall my own initiation into Newtonian dynamics, where *force* was first given precise meaning. (Although Hooke’s Law was known, Newton gave no spring-based operational definition of force in *Principia.* Apparently, force measurement from spring extension was not used until the 18th century [24]). Not realizing that “force” had been adapted from common language, my freshman-year response was to conclude that everyone else was using the word incorrectly.

Newton gave eight technical definitions before stating his three Laws of Motion. Unlike Thomson’s invention of “thermo-dynamics”, Newton did not invent most of the terminology he used in *Principia.* Four of them were adapted from common usage. Usually ignored in physics texts, all his definitions are worth stating.

Newton defined *mass* (or “quantity of matter”, sometimes “bulk”), and *motion* (or “quantity of motion”; what we now think of as momentum). He also defined *impressed force*, with examples that included what we now think of as impulse (collisions and “percussion”), as pressure, and as force; clearly there was some lack of precision in units; in the end this did not matter because when relevant Newton employed ratios. Newton’s fourth definition also was not really new: “Vis insita [vis inertia], a power of resisting, by which every body, as much as in it lies, endeavours to persevere in its present state, whether it be of rest, or of moving uniformly forward in a right [straight] line.” Among other things this definition permits the dissipation-free inertial “resistance” to changes in motion to be distinguished from true dissipative frictional forces that oppose any motion relative to some medium. Inertia“limits” acceleration, but is not really a force.

Newton’s last four definitions characterize attractive central forces (“centripetal”) and their properties; Newton had magnets and gravity in mind.

### 3.2. Energy and Work

In 1668, Wallis, Wren, and Huygens independently showed that, in one-dimensional collisions, what we now call momentum is conserved. Wallis discussed inelastic collisions, and Wren and Huygens discussed elastic collisions, with Huygens using rules equivalent to momentum and energy conservation. In 1686, in treating collisions, Leibniz introduced *vis viva* (living force) for $mv^{2}$—what we would now call twice the kinetic energy [7,25]. This use of ‘force’ for a quantity proportional to an energy may be the origin of the later usage of ‘force’ in the context of energy.

The modern terms energy, kinetic energy, and potential energy all had to wait years to be defined, even when symbols for them existed. Sometimes an equation used the modern symbol and definition, but not the modern name. In his *Analytical Mechanics* (1788) Lagrange employed the symbol $T=mv^{2}/2$, but did not call it kinetic energy; moreover, he employed the symbol *V* but did not call it the potential energy.

Coriolis employed (1829) the term *quantité de travail* (quantity of work) for $mv^{2}/2$, and Jean-Victor Poncelet around that time, like Coriolis, used *travail mécanique* (mechanical work) for $mv^{2}/2$. The form Fdx was known in mechanics and the form pdv was known in the theory of machines. Jammer states [26] that Coriolis introduced the factor of 1/2 in *vis viva* for mathematical convenience, and that he “coined the word ‘work’ for the product of force and distance.” Thus, for a time ‘work’ meant both work and *vis viva.*

In his papers of 1834 and 1835, Hamilton used the initially unnamed symbol *U*, which is opposite in sign to the modern potential energy: Hamilton wrote $m\ddot{x}=\partial U/\partial x$. He called 2T the “living force” (vis viva), thus using Leibniz’s definition. He noted that dT=dU, so integration gives T=U+H, where the constant *H* is not given a name. He wrote $V=2\int_{0}^{\infty }Tdt$ and called it “the accumulated living force, called often the action”. Midway through his 1834 paper, Hamilton called *U* the force function (not the potential energy) [27,28].

In 1847, Helmholtz established mathematically, for central forces, the concept of conservation of mechanical energy, which, strange to the ear of the modern physicist, he (and, it appears, most others of the time) called conservation of force. He also wrote “For the sake of better agreement with the customary manner of measuring the intensity of forces, I propose calling the quantity $mv^{2}/2$ the quantity of *vis viva*, by which it is rendered identical with the quantity of work.” Thus he redefined *vis viva* as did Coriolis, thus helping to establish the modern definition. (Helmholtz considered a mass initially at rest and falling under gravity *g* by a height *h*, so $mv^{2}/2=mgh$.) However, he used the term *tensions* for what we would now call the potential energy, “an increase in vis viva...can only be expended by a decrease in tensions” [29,30].

Young’s 1802 term “energy” was not adopted for some fifty years [21], when in 1853 Rankine wrote: “The law of the Conservation of Energy is already known, viz., that the sum of all the energies of the universe, actual and potential, is unchangeable” [31]. Rankine’s “actual energy” was supplanted in 1862 by Thomson and Tait’s “kinetic energy”, but his “potential energy” was adopted [7,32].

### 3.3. Temperature

Early concepts of temperature and heat were imprecise and, not surprisingly, the two often were conjoined. Around 1592 Galileo invented a *thermoscope,* a non-calibrated device using a water-filled container open to the air containing bulbs of varying density, the “temperature” determined by the lowest bulb that floated. (Basically it was a densitometer, as used in pre-encapsulated lead-acid cell automobile batteries to determine the relative amount of sufuric acid in the electrolyte.) In 1612, the physician Santorio added a numerical scale.

The term “thermometer” (thermo for heat, meter for measure) appeared in print in 1626, in a book on mathematical recreations. According to the Oxford English Dictionary, Puzzle Number LXIX described a device like that of Galileo: “Of the Thermometer: or an instrument to measure the degrees of heat and cold in the aire.”

Such open devices were also sensitive to pressure (early thermometers were often referred to as “weather-glasses”, and used in meteorology); enclosed devices followed by the 1630s, and different fluids (such as alcohol and mercury) were also employed. In 1665, Boyle, Hooke, and Huygens proposed calibration using a single fixed point (freezing or boiling of water) and then thermal expansion relative to that, but no agreement was reached. “Temperature” was first used in 1670 by Boyle [33]. Shortly after 1670 numerous people proposed using two fixed points with a standardized interval. Fahrenheit developed his scale around 1710; the Celsius scale was developed around 1742 [34].

### 3.4. Heat

Heat received its first quantitative definition through calorimetry, a method used but not named by Joseph Black, who measured *relative* specific heats (he used heat ‘capacity’) and discovered latent heat (1761) at the ice-water transition. In the language of that time, “sensible heat” caused temperature change, whereas “latent heat” caused (then as now) melting or boiling with no change of temperature (hence hidden, or “latent”). Black never published, but as a famous teacher his work was disseminated by his students. Only in 1780 did (the non-explorer) Magellan introduce “specific heat” [10].

*Calorimetry* was defined by Lavoisier and Laplace in 1783, from heat (calor) and measure (metry), when they invented the ice calorimeter [35]. This device, which measured the amount of ice in an ice-water mixture that was melted by a given amount of heat, had a much greater precision than previous methods. The (large) calorie (heat to raise one kilogram of water by one degree Celsius) was not defined until 1824, by Clément, a teacher of Carnot; the (small) calorie not until 1852 [36].

In what has been described as a “classic” manual on steam-engine theory, and intended for practicing engineers, but likely too abstract and mathematical for its purpose, Rankine defined the term “adiabatic” in 1859 [37].

*Entropy* was defined in 1865 by Clausius [38]. Unlike the other extensive variables of volume and particle number, entropy cannot be determined by direct measurement; there is no “entroscope”, no less an “entrometer”.

## 4. Friction, Fire, Phlogiston, Caloric

Early humans surely were aware that rubbing their hands together would warm them, and that sufficient and vigorous enough rubbing together of sticks would ignite kindling and thus produce fire. In modern terminology, rubbing converts mechanical energy into heat, and rubbing to the point of ignition involves that conversion of energy and the ignition of fire. No self-respecting natural philosopher (physicists in former times) could avoid thinking about the nature and relationship between heat and fire, and perhaps even have a hazy idea of temperature.

Before it was recognized that work and heat are both quantities of the same type, there were at least three primary trains of thought about their relationship:

(1) The non-quantitative idea that work produces heat; non-quantitative because neither work nor heat had any precise meaning. Fire was not in the picture. This is exemplified by early observations of Francis Bacon (1617), and later by Newton, Leibnitz and others [7].
(2) The conjoining of fire and heat, in the late 1600s and early 1700s, with the idea that a substance called *phlogiston,* having mass (weight), is released on burning. There were also multiple, less chemically-based *fire* theories, with massive or massless particles of fire entering an object as it heats.
(3) The separation of fire and heat, in the *caloric* idea of a massless conserved heat fluid, and the placement of fire within the domain of chemistry and chemical reactions.

Eventually, of course, it was recognized that work and heat are both forms of energy (to extend the language of Aristotle, in the same *category*); this became the basis of thermodynamics.

### 4.1. Gases

In 1660, Boyle, with the essential technical assistance of Hooke, implemented his design for the vacuum pump. This enabled studies of gases at various pressures, which played an important role in understanding heat. It led to the discovery, independently by both Boyle and Mariotte, that pv is a constant at fixed temperature, and led to the idea that gases are “elastic” fluids.

In 1699, Amontons studied the behavior of air under temperature and pressure changes, for fixed volumes (and the range of temperatures he was able to study), finding that Δp/p∼ΔT. (This is expected for small variations of pressure *p*, because the baseline ideal gas temperature $T_{g}$ then does not vary significantly, so the more accurate $\Delta p/p=\Delta t/T_{g}$ is approximately satisfied.) In 1703, he developed a constant volume thermometer based on pressure measurement. Extrapolating to zero pressure he obtained an absolute zero of temperature at about $240{\;}^{\circ }$C below the freezing point of water.

Following the recognition of distinct gases around 1775 by Lavoisier and others, Amontons’s old results were made more precise by Gay-Lussac (1808) and by Dalton (1802). They found, for a given amount of gas at fixed volume and *t* in Celsius, that p/(a+t) is constant, where a≈262 ${}^{\circ }$C [7]. This led to the development of gas thermometry [7].

### 4.2. Heat as Fluid, Phlogiston and ‘Fire’

By the early 1700s two distinct concepts had been developed to deal with heat and fire.

In 1667, Becher posited the conserved substance *phlogiston,* to be contained within combustible objects and released during combustion as fire. Thus phlogiston related both to chemistry and to heat. Stahl, in the early 1700s, further developed the idea that phlogiston has weight, or *mass.* However questionable in retrospect was his idea of phlogiston, Stahl made the very real discovery that metals are combustible, yielding a “calcinated” form (to the modern, an oxide) that weighs more than the starting material. In the hands of Lavoisier, this discovery was later used to reject the idea of phlogiston.

The distinct conserved substance *fire* was, in contrast, posited to be a weightless, or *massless,* fluid. It was used by Boerhaave, at Leyden from 1702, to explain heat, light, flame, and combustion. For Boerhaave, the motion of the particles of matter caused the sensation of heat, but ‘fire’ caused that motion [10]. Fahrenheit’s thermometers figured in Boerhaave’s chemistry and medicine lecture demonstrations and in his influential textbooks [39]. Boerhaave was aware that, when different materials at different temperatures were mixed, their common final temperature was not in relative proportion to their weight. However, he did not have a concept like that of specific heat. On the other hand, he appreciated the effects of surface area on heating and cooling [7].

### 4.3. Heat as Fluid—Caloric

As noted earlier, by 1760 Black had developed the idea of quantity of heat, and later discovered latent heat (for both the water-steam and ice-water transitions). For water-steam he compared the heat required for water at boiling to actually boil off, and for that same heat determined the temperature change it would produce for lower temperature water. Although he knew that calorimetry experiments were readily explained using the idea of heat as a conserved fluid, Black remained cautious about accepting this idea; he was aware of the alternative ‘vis viva’ theory of heat.

In 1777, based on very accurate measurements of weight, Lavoisier published extensive studies on the combustion (oxidation) and calcination (reduction, or de-oxidation) of metals; the metallic samples respectively gain and lose weight in oxidation and reduction [40]. This work marks the introduction of modern chemistry, with the identification of 33 chemical elements and the promise of more. Acknowledging a debt to Boerhaave and to Franklin, Lavoisier explained the chemistry of chemical reactions using ‘air vital’ (oxygen), and the associated heat by a conserved fluid (to become ‘caloric’ by 1783), which he called the ‘matter of fire’ (*matière de feu* or *fluide igné*) [10,40,41]. In combustion, the inflammable substance had a greater affinity for the ‘air vital’ than for the ‘matter of fire’, so the chemical reaction occurred with the release of ‘fire’. ‘Fire’ existed in two states: free (which gave the sensation of heat) and combined (with no such sensation of heat). The amount of free fire within an object gave a measure of its hotness. At this point temperature and heat were still conjoined because the effects of thermal conduction had yet to be unraveled.

That same year, Lavoisier noted that the ‘phlogiston principle’ was not consistent with the weight gain, rather than loss, for ‘calcined’ metals. This became the nail in the coffin for the phlogiston theory [42,43,44]. In 1783, Lavoisier and Laplace invented the ice calorimeter [35]. *Memoire sur la Chaleur* proposed a conserved heat fluid, caloric, like the electric fluid proposed by Franklin (1751) and the magnetic fluid proposed by Aepinus(1759) [10]. It advocated use of the ice calorimeter to measure specific heats, and noted that the specific heat can depend on the material and on its temperature. It also noted that thermal expansion, especially for gases, can lead to the apparent disappearance of heat. (This was a foreshadowing of energy conservation; as noted above, it was later exploited by Mayer to obtain a value for the mechanical equivalent of heat.) They wrote down and employed the equations for mixtures in calorimeters, which used conservation of caloric. However, their paper did not take sides on the material vs. the dynamical (vis viva) theory of heat, noting that vis viva can more easily explain friction, whereas caloric can more easily explain calorimetry [35,45]. “Perhaps both hypotheses are true simultanously.” Caloric was a conserved quantity, passing from one material to another, and provided a natural explanation for quantitative specific heat and latent heat measurements using calorimeters. The concept of caloric, although eventually abandoned, was an organizing principle for some 70 years.

A related theory of a ‘fire’ substance was developed by Cleghorn in 1779. The ‘fire’ was self-repulsive, but attracted to ordinary matter. The amount of ‘fire’ determined the hotness of an object. His idea of fire particles was very similar to that of *caloric.* When adiabatic heating and cooling came to be studied, Cleghorn invoked the inward and outward flow of massless conserved particles of ‘fire’ (*igneae particulae*) [10]. Despite other related theories, eventually caloric prevailed.

The related topic of thermal conduction did not receive serious study until around 1800. It was then recognized that, just as different metals conduct electricity differently, they might also conduct heat differently. This led to time-dependent experiments giving results that depended also on the specific heat. Biot (1804) set up steady-state thermal conduction experiments to isolate *thermal conduction;* these later were the basis of Fourier’s 1823 theory of heat flow. Because of difficulties obtaining reproducible electromotive forces from batteries of voltaic cells, analogous measurements of electrical conductivity were not performed until 1825. Ohm then exploited the recently-discovered and stable temperature-induced voltages (Seebeck effect) to discover his well-known laws, and to carry over Fourier’s theory of the flow of heat to the flow of electricity.

Heat transfer by (electromagnetic) radiation received intensive study in Switzerland by 1791. De Saussure and Pictet established that a hot (cold) object at the focus of a mirror caused heating (cooling) at the focus of a conjugate mirror. This led to Prévost ’s “theory of exchange”, whereby objects are always emitting and absorbing radiation. These workers tended to favor the material (caloric) theory of heat, but were aware of the difficulties of explaining frictional heat [7].

In 1815, in order to improve Newton’s theory of sound, which underestimated the sound velocity *s* in air, Laplace employed caloric *Q* as a function of *p* and *v* [46]. In modern terminology, he treated Q(p,v) as a state function. This is not the case. However, small changes dQ can be treated in this way, as long as they are not treated as true differentials. With *Q* Laplace could define distinct and measurable specific heats *c* at constant *v* and *p* [5]. Then, for simplicity using *T* for $T_{g}$ and taking a single mole N=1, so pv=RT,

(6) $$\begin{array}{ccc} dQ & = & \frac{dQ}{dp}{|}_{v}dp+\frac{dQ}{dv}{|}_{p}dv=\frac{dQ}{dT}{|}_{v}\frac{dT}{dp}{|}_{v}dp+\frac{dQ}{dT}{{|}_{p}\frac{dT}{dv}|}_{p}dv\\ & = & c_{v}\frac{v}{R}dp+c_{p}\frac{p}{R}dv. \end{array}$$

From the idea that during a sound wave oscillation heat did not have time to leave a compressed or expanded region of air, so dQ=0 (the adiabatic condition), Laplace could relate dp and dv. This enabled him to explain *s* phenomenologically in terms of the ratio $\gamma =c_{v}/c_{p}$.

### 4.4. Heat as Motion—Vis Viva

From the beginning of modern dynamics, the idea of heat as motion (‘vis viva’) had significant support. To account for slowing due to friction, Leibniz claimed that heat consisted of the random motion of the constituent parts of matter. In later editions of his *Opticks,* Newton suggested the related idea that heat is due to the rapid motion of parts of bodies. He also suggested a kinetic theory of evaporation, and a kinetic theory for the increase of pressure with temperature of gases. Nevertheless, Newton did not consistently use kinetic theory ideas, rather explaining Boyle’s Law by invoking a force law that decreases with separation [7].

Around 1735 Daniel Bernoulli developed a kinetic theory argument for gas pressure. It gave the pressure inversely proportional to the volume, as in Boyle’s law, and the theory is much as we teach today. One reason it did not persist is the lack of evidence for atoms, which did not appear until Dalton’s work around 1800 [7].

Despite the success of the idea of conserved caloric as an explanation for heat, frictional heating remained distinct from putatively conserved caloric. Nevertheless, the heat-producing friction experiments of Rumford (1798) [47] and of Davy (1799) [48]—cited by Thomson in 1851 to the detriment of the caloric theory—could be interpreted in the framework of the caloric theory. That is, it could be argued that caloric, like the fluids of Franklin’s electricity and Aepinus’s magnetism, is both low in mass (“imponderable”) and extremely abundant. Thus it was possible for scientists to continue to retain both the material and mechanical conceptions of heat, using whichever was most convenient for a given application [7,10].

## 5. On the Steam Engine

By the time of the first steam engine (Savery, 1698), Britain had exhausted most of its forests near population centers, so to support its population’s energy needs it employed coal. Although Savery’s device was terribly inefficient, it was the best available at the time [7,49]. In this piston-less device, a boiler produced steam, which by a valve could enter a large metal cylinder connected to a water reservoir below it. After steam filled the cylinder, a valve was closed to the atmosphere, and then cold water was poured over the cylinder, thus cooling the steam and decreasing its pressure, and therefore drawing up water from below. After removing the drawn-up water, another valve was opened to release the now-cooled steam from the cylinder to the atmosphere. This drew water to a height of 32 feet—the atmospheric pressure of water. The procedure was then repeated.

This design was significantly improved in 1712 by Newcomen, whose engine was not limited by atmospheric pressure. He employed a massive rocker on a pivot through its midpoint. On one side of the rocker was a boiler, above which was a cylinder with a piston; on the other side of the rocker was a vertical rod. First the boiler-heated steam drove the piston and the cylinder upward, so the vertical rod on the other side was driven downward. Next, cold water was sprayed on the outside of the cylinder, the resultant cooling decreasing the pressure in the cylinder and thus driving it downward and the rod upward. To optimize the Newcomen engine, Smeaton systematically varied (‘cut-and-try’) one parameter at a time. This improved the efficiency by a factor of two, with no fundamental design changes [50].

In the winter of 1763/64, the “mechanic” Watt repaired a demonstration Newcomen engine at the University of Glasgow [7]. After the repair, Watt studied how to improve it. Recognizing the large heat loss on cooling the metal boiler-cylinder (the steam vessel), Watt tried a wooden cylinder; this decreased the heat loss, but the wood did not last. In 1765, on the cylinder side Watt added a second heat reservoir (the condenser) at a lower temperature, into which the hot steam was released for cooling. Like that of Savery, the boiler-only Newcomen engine may be thought of as a simple *fire* engine (the wood or coal fire almost directly heating the water). However, Watt’s addition of the condenser made it a true two-thermal reservoir *heat* engine [7]. This practical innovation became a crucial part of Carnot’s 1824 analysis of its efficiency.

Further, in 1769 Watt recognized the ‘expansive principle’, which economized on fuel by opening the condenser valve a bit early; the piston would continue to perform work by moving under its own inertia. Watt made numerous other technical advances, such as his centrifugal ‘governor’ to regulate the admission of steam into the cylinder.

In 1792, Davies Gilbert (aka Davies Giddy), a friend of Watt, recognized that the work pdv done by the steam is the area under a *p* vs. *v* curve [7]. In 1796, John Southern, one of Watt’s assistants, developed a method to write this information from a pressure read-out [7]. This led to Watt’s “indicator diagram”, a trade secret until 1822. That same year Watt wrote that, years before, after learning from his own work that steam can heat six times its own weight in water to boiling, and being puzzled by that fact, he consulted with Black, also at Glasgow. The latter explained the concept of latent heat and related topics. Watt noted that gaining this knowledge did not suggest to him improvements to the steam engine, but they “certainly conduced very much to facilitate the progress of my inventions...” [51].

This concludes our pre-history of thermodynamics.

## 6. Carnot

Carnot’s short book (1824), begins “Everyone knows that heat can produce motion ...To heat also are due the vast movements which take place on the earth” [8,12,13]. Thus, in addition to understanding the steam engine, even earlier motivations for developing thermodynamics arose in studies of meteorology and the atmosphere. For example, invoking thermal expansion of the atmosphere, Halley discussed the origin of the trade-winds, noting that the sun provides maximum heating at the equator [7]. In this current time of global warming, increased temperatures lead to increased wind velocities and to transfer of weather from one part of the globe to another. Without thermodynamics, a result of Carnot’s work, there would be no hydrodynamics of heated fluids, and no theoretical structure for thermal stabilities.

### 6.1. Analyzing the Efficiency of Heat Engines

Sadi Carnot’s father was “hero of the Republic” Lazare Carnot, the general who defeated the royalist (counter-revolutionary) armies after the French Revolution. But Lazare also wrote on mathematics and mechanical machines (powered by wind, water, and horses). He emphasized that, for maximum efficiency, connections between moving parts should be made as smooth as possible (no percussion—no banging, no sound, no dropping); that is, one should minimize the mechanical impedance mismatch. Watt’s ‘expansive principle’ is in this category [7].

Sadi, educated at the École Polytechnique, like many other French engineers took an interest in British progress on steam engines during the years when the Napoleonic wars limited communication. We can recognize that, for the steam engine, the analog of his father Lazare’s avoidance of mechanical engine mismatch was Sadi’s avoidance of thermal mismatch. This meant no direct contact between objects at different temperatures. It led him to conceive of what we now know of as the Carnot cycle, with two isothermal legs (of the working medium with the hot and cold reservoirs) and two adiabatic legs (to permit the working medium to heat or cool in thermal isolation, before or after connecting to the hot or cold reservoir). Working within the context of the caloric theory he developed this cycle in his 1824 publication *On the Motive Power of Fire* [12,13].

In this work, Carnot made other important and essential contributions: (1) the remarkable conceit of the reversible heat engine; (2) the imaginative use of the principle that there can be no perpetual motion; (3) the realization that, in computing the efficiency for production from heat of motive power (work output), one must include both the positive work done when the working medium contacts the hot reservoir H, and the negative work done while it contacts the cold reservoir C. (He did not consider the work done during the adiabatic expansion and contraction processes; however, for the ideal gas they cancel). Using modern notation for heat *Q* and net work *W*, Carnot in effect took the thermodynamic efficiency to be W/Q.

Carnot considered two reversible engines (based on *any* cycle) operating between the same two temperatures with different efficiencies. To illustrate his reasoning, let two such heat engines absorb 10 units of heat, but one engine produce 2 and the other 3 units of work. Running the more efficient one forward (to produce 3 units of work) and the less efficient one backward (to use 2 units of work), gives a net work production of 1 unit, with no net change in caloric at either the hot or cold reservoir. Carnot rejected this possibility of work production with no change in heat content; it was even less acceptable than simple perpetual motion. He therefore concluded that all reversible heat engines must have the same efficiency, a result that, in the hands of Clausius and later Thomson, morphed into the two forms of Second Law of thermodynamics. (Carnot employed the word ‘primatif’, or ‘primitive’, to mean ‘initial’, in the sense of a cyclic process. This perhaps-confusing usage of ‘primative’ persisted in some later works).

### 6.2. Difficulties with Carnot’s Analysis

Although his definition of efficiency survives the transition to energy conservation, and even though he realized that the net work must be computed [7], there are difficulties with Carnot’s work. Therefore we present no details of his analysis; neither numerics from his text, nor analytical arguments from a multi-page footnote.

For the ideal gas he uses the reasonably accurate pv∼(t+267), with *t* in Celsius. He considers only the (by assumption) conserved heat provided by the hot reservoir; this agrees with the calculation of the modern thermodynamic efficiency $e=W/Q_{H}$, because $Q_{H}=Q$.

A major difficulty for Carnot’s analysis is that for the specific heats at constant volume $c_{v}$ and at constant pressure $c_{p}$, Carnot relied on the specific heat experiments of Delaroche and Bérard, whose authors incorrectly thought that their measurements implied that the specific heat of air depends on pressure [7]. Thus Müller writes of Carnot: “This misconception [of caloric], and the false information which Carnot had about the specific heat of gases and the latent heat of water vapour, invalidates much of the second half of his paper. He tied himself into knots over the specific heats of gases, which he thinks he can prove to be logarithmic functions of the density when in reality they are constants, independent of both density and temperature” [15].

This small book, published in 1824 when he was twenty-eight—he died of cholera in 1832—was Carnot’s only publication. He had no editor, and likely his only reader was his brother, Hippolyte, who could not help on technical matters. In 1878, Hippolyte placed Sadi’s previously unpublished notes in the care of the Academy of Sciences. These reveal that by 1832 Sadi had recognized the principle of conservation of work and heat, and that he had estimated the mechanical equivalent of heat. He wrote “It would seem, then, that the heat set free [by percussion, banging] should be attributed to the friction of the molecules of the metal ...that is, the heat is set free just where the moving force is expended.” “Wherever there is destruction of motive power there is, at the same time, production of heat in quantity exactly proportional to the quantity of motive power destroyed” [12]. He also had proposed experiments to test these ideas, ideas not to be discussed by others for nearly twenty years [5,12,13,].

Many translations include these notes as Appendices [12,13]. We give no further details of Carnot’s work; it his conceptual advances that for our purposes are most significant. For more detail see Ref. [7]. Curiously, the Sadi Carnot so well-known in thermodynamics is far less well-known to the French general public than his similarly named nephew, who served as President of France.

## 7. Clapeyron

Clapeyron (1834) expands on Carnot’s work [14]. He does not number his equations, and sometimes writes an equation partly in the text and partly in an inset without an equals sign. This sometimes makes his paper difficult to follow and to describe [12]. For clarity we therefore employ brackets [] both to indicate where we have added implicit terms, and to aid the reader to identify the original unnumbered equations by section and order in the section.

Clapeyron gives the first graphical representation of the Carnot cycle in *p*-*v* space (Watt’s ‘indicator diagram’). Had he known to employ ‘heat absorbed minus work done’ as conserved, his approach would have—except for entropy—led to modern thermodynamics. Most of his detailed results are correct. He discusses the ideal gas before steam.

We discuss his Sect. III on the ideal gas, and his Sect. IV on the vapor-liquid (steam) system, including a derivation of what is now known as the Clausius-Clapeyron equation. He also considers other single phase systems, such as iron, in his Sect. V.

### 7.1. Ideal Gas and Heat Function *Q*

In his Sect. III Clapeyron considers the ideal gas as his working medium, with units, as for Carnot, taken to satisfy

(7) pv=R(267+t).[3.3]

Clapeyron gives figures of *p* vs. *v* for the ideal gas and for steam, but he labels the axes as *Y* and *X*.

Within the text Clapeyron gives, for two thermal reservoirs at *t* and t−dt, and volume change dv, the net work (motive power)

(8) $$\left[dW=dpdv=\right]\frac{R}{v}dtdv;\;\left[3.4\right]$$

He uses “quantity of action” for work, so we here we include [dW=dpdv] to produce an equation. This equation is valid for an infinitesimal Carnot cycle, where the legs in *p*-*v* space, although tilted relative to one another, are straight.

Clapeyron next assumes that there is a well-defined heat function Q(v,p). This follows Laplace’s considerations on the speed of sound in air [46]. Hence, as in (6),

(9) $$dQ=\frac{dQ}{dv}dv+\frac{dQ}{dp}dp.\;\left[3.5\right]$$

By the caloric theory Clapeyron needs only consider dQ for one of the isothermal legs of the cycle; by definition, for the adiabatic legs dQ=0.

For *t* constant, as applies when the working medium is in contact with either thermal reservoir, Equation (7) yields

(10) $$dp=-\frac{p}{v}dv.\;\left[3.6\right]$$

Hence, in a notation with implicit partial derivatives, Clapeyron obtains

(11) $$dQ=\left(\frac{dQ}{dv}-\frac{p}{v}\frac{dQ}{dp}\right)dv.\;\left[3.7\right]$$

From (8) for the motive power and (9) for the heat, Clapeyron obtains the Carnot-determined efficiency

(12) $$\left[\frac{dW}{dQ}=\right]\frac{Rdt}{v\frac{dQ}{dv}-p\frac{dQ}{dp}},\;\left[3.8\right]$$

where we include [dW/dQ=] to form an equation. Like Carnot, Clapeyron implicitly assumes that heat and work are proportional. By Carnot’s reversibility argument, dW/dQ should depend only on *t* (and dt), so as done shortly, the denominator of (12) may be written as CR, with *C* an unknown function only of *t*.

### 7.2. Ideal Gas and Carnot Efficiency

Since t∼pv by (7), with CR≡F one may also write the denominator of (12) as

(13) $$v\frac{dQ}{dv}-p\frac{dQ}{dp}=F\left(pv\right),\;\left[3.9\right]$$

where F(pv) is an unknown function.

Assuming that *Q* is a true function of *p* and *v*, then the solution is, for another unknown function f(pv),

(14) Q=f(pv)−F(pv)log(p).[3.10]

Using pv∼t, Clapeyron rewrites this as

(15) Q=R[B−Clog(p)],[3.11]

where both B(t)=f(t)/R and C(t)=F(t)/R are unknown functions of *t*. Since there is no such thing as a heat function *Q*, this result is not trustworthy. Nevertheless, Clapeyron applies (15) to obtain three results, two of them valid:

First, by F=CR, (13) becomes

(16) $$v\frac{dQ}{dv}-p\frac{dQ}{dp}=CR,\;\left[3.14\right]$$

so that (12) becomes

(17) $$\frac{dW}{dQ}=\frac{dt}{C}.\;\left[3.15\right]$$

The unknown function C(t), which does not appear in Carnot, is the heat per work per temperature change dt. This result is valid because only dQ was used.

In 1854, Clausius used (17) and (8) to write, in a footnote,

(18) $$\frac{dQ}{dv}=\frac{dQ}{dW}dp=C\frac{dp}{dt},$$

an important result that he employed, as we discuss later, in establishing that the thermodynamic temperature *T* and the ideal gas temperature $T_{g}$ are the same.

### 7.3. Ideal Gas and dependence of *Q* on *v*

Second, from Equation (15) one obtains, for two different pressures at the same *t*,

(19) $$Q-Q^{\prime }=CR\log\left(\frac{p^{\prime }}{p}\right)=CR\log\left(\frac{v}{v^{\prime }}\right).\;\left[3.16\right]$$

Further, with

(20) $$R=\frac{p_{0}v_{0}}{267+t_{0}}=\frac{pv}{267+t}\;\left[3.17\right]$$

one can show that

(21) $$Q-Q^{\prime }=\frac{pv}{267+t}C\log\left(\frac{v}{v^{\prime }}\right).\;\left[3.21\right]$$

(The original equation has a misprint, with (267+t)/(pv) inverted.) Clapeyron observes that this yields a dependence on pressure or volume (as also found by Carnot); Mendoza comments that such a dependence is incorrect [12]. This result depends on *Q* itself, but *Q* is not a state function.

### 7.4. Ideal Gas and Specific Heats

Third, at the end of Sect. III Clapeyron notes that on differentiating Equation (15) with respect to *t* at either constant *p* or *v* one obtains

(22) $$\left[c_{p}=\right]R\left(\frac{dB}{dt}-\frac{dC}{dt}\log p\right)\;\left[3.22\right]$$

and

(23) $$\left[c_{v}=\right]R\left(\frac{dB}{dt}-\frac{dC}{dt}\log p-C\frac{1}{p}\frac{dp}{dt}\right).\;\left[3.19\right]$$

With (20), these lead to a familiar result that Clapeyron does not actually write down,

(24) $$c_{p}-c_{v}=\frac{R}{267+t}C.$$

Because it involves the *difference,* due only to work, this result is valid.

Mendoza comments that these equations make $c_{v}$ and $c_{p}$ individually (and incorrectly) pressure-dependent, and therefore “They are almost the only ones of Clapeyron’s equations for observable quantities which are actually wrong” [12]. This is because (22) and (23) explicitly depend on the heat “function” Q(p,v), which introduces the incorrect dependence on *v* or *p*.

### 7.5. Vapor-Liquid (Steam)

Water, when heated, has appreciable amounts of each of two fluid phases at relatively high and low densities; the gaseous (vapor) and liquid phases, as in steam. Since Carnot gave steam engines little detailed consideration, much of the analysis that follows may originate with Clapeyron.

Clapeyron takes the vapor to have mass density δ (not the modern symbol for a small change) and the liquid to have mass density ρ. If a volume *v* of vapor is formed, then it comes at the cost of liquid volume v(δ/ρ), so the change in system volume dv is

(25) $$\left[dv=\right]v\left(1-\delta /\rho \right).\;\left[4.1\right]$$

Therefore the “quantity of action” (our dW=dpdv) is

(26) $$\left[dW=\right]\left(1-\frac{\delta }{\rho }\right)v\frac{dp}{dt}dt,\;\left[4.2\right]$$

where we include [dW=] to form an equation. With latent heat *k* per unit volume of gas formed, if a gas volume *v* is formed, then the amount of heat produced is dQ=kv (Clapeyron does not use any symbol here), so

(27) $$\left[\frac{dW}{dQ}=\right]\frac{1}{k}\left(1-\frac{\delta }{\rho }\right)\frac{dp}{dt}dt,\;\left[4.3\right]$$

where we include [dW/dQ=] to form an equation. Having demonstrated by Carnot’s arguments that this efficiency is the maximum possible, and material-independent, by [3.15] Clapeyron sets this equal to dt/C, where *C* is the same unknown function introduced earlier for the ideal gas. Thus he obtains

(28) $$\frac{1}{k}\left(1-\frac{\delta }{\rho }\right)\frac{dp}{dt}=\frac{1}{C}.\;\left[4.4\right]$$

With the latent heat *L* as heat per unit mass transformed, so L=k/δ, and volume change per unit mass transformed as Δv=(1−δ/ρ)/δ, this is equivalent to the modern

(29) $$\frac{dp}{dt}=\frac{1}{C}\frac{L}{\Delta v}.$$

Since typically δ/ρ≪1, this may be rewritten as k≈C(dp/dt), which, as Clausius notes, relates the vapor curve to the latent heat.

Clausius in 1851 showed that (for these pre-mechanical equivalent of heat units) C=T, so this 1834 result (28) written as (29) is what we now call the Clausius-Clapeyron equation.

### 7.6. General Case

In Sect. V of his paper, Clapeyron considers the general case of a Carnot cycle, but in this section he (or the type-setter) changes to *T* from *t*. At either thermal reservoir dT=0 and dQ≠0. Then

(30) $$\left[dT=\right]\frac{dT}{dp}dp+\frac{dT}{dv}dv=0,\;\left[5.4\right]$$

(31) $$dQ=\frac{dQ}{dp}dp+\frac{dQ}{dv}dv=dv\left[\frac{dQ}{dv}-\frac{dQ}{dp}\frac{dT/dv}{dT/dp}\right].\;\left[5.5\right]$$

Thus (we introduce dW)

(32) $$\left[\frac{dW}{dQ}=\frac{dp\;dv}{dQ}=\right]\frac{dT}{\frac{dQ}{dv}\frac{dT}{dp}-\frac{dQ}{dp}\frac{dT}{dv}}.\;\left[5.6\right]$$

However, he has argued quite generally in (17) that dW/dQ=dt/C, so he deduces that

(33) $$C=\frac{dQ}{dv}\frac{dT}{dp}-\frac{dQ}{dp}\frac{dT}{dv},\;\left[5.7\right]$$

and therefore that

(34) $$dQ=dv\frac{C}{dT/dp}=-dp\frac{C}{dT/dv},\;\left[5.10\right]$$

or

(35) $$dQ=-dp\;C\frac{dv}{dT}.\;\left[5.11\right]$$

Clapeyron then notes (his italics) that the above equation implies that “*If the pressure supported by different bodies, taken at the same temperature, be augmented by a small quantity, quantities of heat will be disengaged from it, which will be proportional to their dilatability by heat*”, followed by “This result is the most general consequence deducible from this axiom: that it is absurd to suppose that motive force [work] or heat can be created gratuitously and absolutely” [12].

In other words, Clapeyron argues that net work done and the heat input are proportional. Nevertheless, the idea that something is conserved, so that the heat output is less than the heat input, does not yet appear.

## 8. Thomson 1848 and 1849

### 8.1. Thomson 1848

Having read Clapeyron, but not yet Carnot’s difficult-to-obtain book, Thomson argued that from the material-independent thermodynamic efficiency of reversible heat engines operating between the same two temperatures, an “absolute thermometric scale can be founded...” [19]. He also realized that, although the air thermometer was very reproducible, its temperature scale might be a function of some other, truly universal, temperature scale.

As already noted, Thomson was later to introduce a different temperature scale, now known as the Kelvin scale, which is identical to what I have called the ideal gas temperature scale $T_{g}$. His later usage was θ for what in 1848 he called *T*, and *t* for what we call the Kelvin temperature *T*.

Thomson in 1848 defined the scale θ as one where all temperature differences have the same value, as determined by equal “mechanical effect” (net work) for fixed unit heat flow between reservoirs at θ and θ−1 degrees, for all θ. That is, he restricted himself to Δθ=1. Thomson also imposes the two temperatures for freezing and boiling of water (at atmospheric pressure).

With this discrete Δθ=1 definition, Thomson compared this temperature θ with temperature values in degrees Celsius *t* from the air thermometer. Regnault had both accurate values for the air thermometer, and accurate values for the work produced by the steam engine, at fixed volume. Table I of Thomson’s later multi-part 1849 paper presents his results, expressed in terms of mechanical efficiency, for which he there develops the theory. Although the 1848 paper contains no equations, in a reprint of this 1848 paper (footnote dated Nov. 4, 1881), Thomson indicates his reasoning. At Joule’s suggestion, the efficiency $dW/dQ∼1/T_{g}$. To obtain the θ scale using dθ rather than Δθ=1, one equates the forms for the efficiency in θ and in $T_{g}$. This gives $d\theta ∼dT_{g}/T_{g}$, which leads to a logarithmic relationship that we rewrite as an exponential:

(36) $$T_{g}=273\;e^{\left(\theta /100)\log(373/273\right)}.$$

Here θ=0 corresponds to $T_{g}=273$ and θ=100 corresponds to $T_{g}=373$. For this “absolute thermometric scale”, θ→−∞ corresponds to the absence of thermal motion.

In the end the ideal gas scale $T_{g}$ is not only valid, but preferable, due not only to its natural appearance in the ideal gas law, but more fundamentally, because of Clausius’s work in 1854, $1/T_{g}$ is the integrating factor 1/T in dQ/T.

### 8.2. Thomson 1849

In the early 1840s, James Joule, with little initial recognition or support, was developing experimental evidence that heat converts to work, and in a specific and universal ratio. He and Thomson became friends after meeting at the 1847 British Association Meeting at Oxford. Within weeks they met by accident on a tour of Mont Blanc. There they tried to measure the temperature rise of fallen water at a waterfall; unsuccessfully, because of the water spray [52].

Although convinced by Joule’s numerous demonstrations of the mechanical equivalent of heat, Thomson had reservations about the total inter-convertibility of heat and work. In particular, he correctly did not believe, all other things the same, as in a cyclic process, that heat can be *totally* converted to work. This prevented him from accepting conservation of energy to the extent needed to formulate a thermodynamics based on it. In the steam engine and the Carnot cycle only *some* of the heat is converted to work.

In 1849, Thomson wrote that: “When ‘thermal agency’ is thus spent in conducting heat through a solid, what becomes of the mechanical effect which it might produce? Nothing can be lost in the operations of nature, no energy can be destroyed. What effect then is produced in place of the mechanical effect which is lost?” Thomson’s biographer, Silvanus P. Thompson, comments that in this phrase the word ‘energy’, “then unknown in its modern signification, here bursts forth in the intuitive utterance” [52,53].

However, in 1849 Thomson could not push beyond this objection, even to accept that in a heat engine the waste heat is less than the input heat. Nevertheless, he suggests that there may be a need “for an entirely new basis of the theory of heat” [54].

In 1850, Clausius used conservation of energy as that ‘new basis’ of the theory of heat. In 1851, Thomson turned his lemon of an objection into lemonade, making his disbelief that heat can be completely converted to work into his form of the Second Law of thermodynamics. Later Clausius accounted for the “loss” of (useful) “mechanical effect” in developing the concept of entropy and recognizing the principle that it cannot decrease [52].

After discussing an important side work, we discuss that 1849 work by Thomson, which was published in two parts.

#### 8.2.1. The Thomson Brothers and the Ice-Water Transition

Knowing that near the melting temperature the density of water decreases on melting, Thomson was concerned that providing heat to an ice-water mixture would, paradoxically, also cause work to be done as the mixture compressed. His older brother James, an engineer, resolved the contradiction in early 1849 by predicting the new result that, as temperature increases, the ice-water melting pressure decreases, so that on heating more solid appears and the mixture volume can expand without having more work done on the mixture [55].

James reasoned as follows. He noted that for a (ft)${}^{3}$ of water (1.087 of ice) and a (ft)${}^{2}$ cross-section, freezing at pressure *p* (in lb/ft${}^{2}$) corresponds to a stroke of 0.087 ft and work (we call it $W_{1}$) of

$$W_{1}=0.087\;p\;\mathrm{ft}-\mathrm{lb}.$$

To remain on the ice-water line this also must correspond to a temperature change of ice by *t* units, which for this system is 4925t “thermic units” and *t* in Celsius. He wrote that one “thermic unit” is equivalent to 4.97 ft-lb. (It appears that James uses Regnault’s data to extract the mechanical equivalent of heat.) Hence

$$W_{2}=\left(4925\right)\left(4.97\right)t.$$

Setting $W_{2}=W_{1}$ he obtains, with atmospheric pressure $p_{\mathrm{atm}}$,

(37) $$t=0.00000355\;p,\;\mathrm{or}\;t=0.0075\;u\;(u=p/p_{\mathrm{atm}}).$$

This does not quite have the form of the Clausius-Clapeyron equation. Although *t* here is a change in temperature, *p* is a pressure, not a pressure change.

In a separate paper, William Thomson reports his observation of the predicted effect [56]. For u=16.8, or 16.8 atmospheres of pressure, the pressure change $\Delta p=p-p_{\mathrm{atm}}\approx p$, so (37) approximately gives Δt vs. Δp. William measured a decrease in freezing point of 0.232 F; this compares well with 0.227 F, as follows from the above equation on converting from C to F. Neither Thomson appears to have been aware that Clapeyron’s paper already contained this prediction. Cardwell notes that to Joule the heat was converted to work, but to Thomson, still accepting caloric, the heat was “squeezed out”: “Carnot’s theory was not merely saved, it had received something like a triumphant vindication” [7].

#### 8.2.2. Thomson’s 1849 Paper Itself

Thomson (William) organizes with numbered paragraphs. The Introduction is very general, with three numbered paragraphs. Part I (paragraphs 4–45) is “On the nature of Thermal agency, considered as a motive power.” It first considers the Seebeck effect (temperature producing an electric current, permitting us “to conceive of an electro-magnetic engine supplied from a thermal origin”), but quickly moves on to Carnot’s work. Part II (paragraphs 46–51) deals with making “thermal agency available as a source of mechanical effect, by means of the expansions and contractions of bodies.” He then illustrates the Carnot cycle with a *p*-*v* diagram, and analyzes the Carnot cycle in detail.

Unlike Clapeyron, Thomson discusses the steam engine before the ideal gas. For steam the pressure depends primarily on temperature and little on volume. He writes, for the net work *W* done in a cycle, or “mechanical advantage”, or “mechanical effect”, or “duty”, or *M*,

(38) $$\left[M=\right]\int_{p_{2}}^{p_{1}}\xi dp,\;\left[20.1\right]$$

where ξ is the fluid to vapor volume change (moderns might call it Δv) across the cycle. He considers heat transfer *H* from the hot reservoir to the cold reservoir in this heat-conservation-based work (this differs from Laplace and Clapeyron’s use of *Q* for heat) and takes latent heat *k* “in a unit [volume] of saturated steam at temperature *t*”. With σ<1 the relative density of steam (a gas, or vapor) to water (a liquid), if volume $v_{L}=\sigma H/k$ of water is heated by amount *H*, then it will produce a volume $v_{v}=H/k$ of steam. Explicitly, with $\xi =v_{v}-v_{L}$,

(39) $$\xi =\left(1-\sigma \right)\frac{H}{k}.\;\left[20.2\right]$$

Thomson takes upper and lower temperature reservoirs to be at *S* and *T*. For small S−T, so dp≈(dp/dt)(S−T), he finds that

(40) $$M=\frac{1-\sigma }{k}\frac{dp}{dt}H\left(S-T\right).\;\left(2\right)$$

This echoes what we have labeled Clapeyron’s [4.4], or (27).

Thomson next considers the air engine. Treating it as an ideal gas Thomson writes, using pressure $p_{0}$ and volume $v_{0}$ at t=0,

(41) $$pv=p_{0}v_{0}\left(1+Et\right).\;\left[26.1\right]$$

Here $E\equiv a^{-1}$ is an inverse temperature, where *a* is the temperature offset between the Celsius and Kelvin scales, taken much earlier as 262 by Amontons, as 267 by Carnot and by Clapeyron, and somewhat later as 273 by Clausius.

With S−T=τ, Thomson then considers a small Carnot cycle (τ≪S,T and dv≪v) that is a slightly distorted rhombus in *p*-*v* space. With volume changes ±dv along the upper and lower isothermal legs, the corresponding pressure changes are ∓pdv/v. In addition, along the high and low volume adiabatic legs there are volume and pressure changes ±ϕ and ∓ω. Without solving for ϕ and ω individually, he finds a combination that is precisely what is needed to obtain the net work. (We have verfied the algebra.)

Thomson then obtains

(42) $$M\left[\approx \xi dp\right]=\frac{Ep_{0}v_{0}}{vdq/dv}H\tau ,\;\left(3\right)$$

where in an isothermal process dq is the heat absorbed, for a given dv. By Carnot, for a given *H* this *M* must be the same for the air engine as for the steam engine.

Thomson’s (4), not shown, involves an integral for finite heat *q* and temperature difference τ; his (5), also not shown, includes the result for the steam engine. Thomson then defines what he calls ‘Carnot’s coefficient’

(43) $$\left[\frac{M}{H\tau }=\right]\mu =\frac{\left(1-\sigma \right)}{k}\frac{dp}{dt}=\frac{Ep_{0}v_{0}}{vdq/dv}.\;\left(6\right)$$

This echoes what we have labeled Clapeyron’s [3.15], or (17).

By Carnot, μ can only be a function of temperature, and thus does not change in isothermal processes. Thomson’s Equation (7) is the more precise $M=H\int_{T}^{S}\mu dt$, but μ(t) is as yet unknown. (With *J* the mechanical equivalent of heat, $\mu \to C^{-1}=J/T$, and (1−σ)/k→Δv/(L/J), the first equality in (43) becomes the Clausius-Clapeyron Equation (29)).

Thomson goes on to use Regnault’s steam data at a given air data temperature *t* in Celsius to compute μ. Not knowing the relation between density and pressure for the vapor in the (two-phase steam), he assumes that the vapor is an ideal gas. The result is his Table I. Clausius’s later and more complete treatment did not make that ideal gas assumption. Ref. [11] provides a detailed discussion.

#### 8.2.3. Thomson’s Four-Part Appendix

*Part I.* This deals with the values of μ derived by Carnot and Clapeyron from observations on air and on some vapors like steam. Thomson notes “slight discrepancies which must be owing to the uncertainty of the experimental data”. In what follows, we emphasize the theory.

*Part II.* This considers the heat produced by compressing air. By rearranging (43), in paragraph 46 Thomson finds, for the ideal gas, that

(44) $$dq=\frac{Ep_{0}v_{0}}{\mu }\frac{dv}{v}.\;\left(8\right)$$

At fixed temperature *t*, on integrating over the compression process from *V* to $V^{\prime }=V-dV$ he obtains for the change in the heat “function” the value (using the Naperian logarithm)

(45) $$Q\left[=\int dq\right]=\frac{Ep_{0}v_{0}}{\mu }\log\frac{V}{V^{\prime }}.\;\left(9\right)$$

He then concludes that, at constant *t*, “Equal volumes of all elastic fluids, when compressed to smaller equal volumes, disengage equal quantities of heat,” which he comments is a theorem of Carnot, previously noted experimentally by Dulong. This *Q* is a change in heat, and as such may be compared with what we have called Clapeyron’s [3.21], or (21); the comparison is consistent with Clapeyron’s *C* being Thomson’s 1/μ, but Thomson does not call attention to this.

Noting that Joule had performed experiments measuring, for the compression of air at fixed reservoir *t*, the heat produced by a given amount of work, Thomson then turns to the corresponding work done in an isothermal process (e.g., at fixed *t*). He finds that $dW=pdv=p_{0}v_{0}\left(1+Et\right)\left(dv/v\right)$ integrates to the in-line equation

(46) $$\left[W=\right]p_{0}v_{0}\left(1+Et\right)\log\frac{V}{V^{\prime }}.$$

He then obtains, at fixed *t*, from (45) and (46), that

(47) $$\frac{W}{Q}=\frac{\mu (1+Et)}{E}.\;\left(11\right)$$

Equation (47) is distinct from the efficiency of a complete Carnot cycle because *W* means only the work on isothermal compression, not the net work in a Carnot cycle.

Thomson notes that this ratio is the same for all gases at the same temperature. In fact, this ratio is the mechanical equivalent of heat. Had Thomson used Joule’s value for the mechanical equivalent of heat, for which Thomson used the symbol *J*, and set W=JQ he would have derived $\mu =J/T_{g}$, a result that in his 1851 paper he wrote had in 1848 been “suggested” to him in a letter from Joule. One wonders: if Thomson knew of Joule’s “suggestion” in 1848, why did he not apply it in 1849? This indicates that Thomson did not accept Joule’s “suggestion” at that time. This point is relevant to an exchange of letters between Clausius and Thomson in 1856 (Section 14)

*Part III.* Here Thomson considers the specific heats of (ideal) gases. By (44), the difference between $c_{p}-c_{v}$ (Thomson’s B−A) is given by the additional term ${\left(dq/dt\right)|}_{p}=\left(dq/dv\right){{|}_{p}\left(dv/dt\right)|}_{p}$. This requires using (41), so that (44) leads to ${\left(dv/dt\right)|}_{p}=\left(p_{0}v_{0}/p\right)E$. Then

(48) $$\frac{dq}{dt}{|}_{p}=\frac{Ep_{0}v_{0}}{\mu v}\frac{p_{0}v_{0}}{p}E=\frac{E^{2}p_{0}v_{0}}{\mu (1+Et)}.$$

This leads to

(49) $$c_{p}=c_{v}+\frac{dq}{dt}{|}_{p}=c_{v}+\frac{E^{2}pv}{\mu {\left(1+Et\right)}^{2}},\;\left(12\right)$$

where we include a dimensionally necessary factor of *v* likely omitted by the printer. Since pv/μ has units heat times temperature, and $E^{2}$ has units temperature${}^{-2}$, the second term indeed has the expected units of heat over temperature. Since $pv,\mu^{-1}∼\left(1+Et\right)$, as expected the last term in (49) is a constant. This is to be compared to (24), which is implied by Clapeyron’s work.

*Part IV.* Here Thomson compares the relative advantages of air-engines (ideal gas) and steam engines, and of water wheels. He notes that with water wheels the fall of water can be converted completely to work if done properly (avoiding splattering, etc.), but this cannot be done with air-engines or steam-engines. Further, noting that air engines (at that time) operated at a higher temperature than steam-engines, with waste heat at about the same temperature, he observes that air engines can be more efficient than steam-engines. We note an analogy to the efficiency of falling water that reaches its lowest value, and thus 100% efficiency, in a succession of discrete steps: consider a sequence of thermal reservoirs with waste heat being re-used at the next lowest temperature reservoir until one reservoir is practically at zero temperature.

*Part V.* In “On the Economy of actual Steam-Engines” Thomson considers steam engine efficiency in theory and in practice. He notes that the theoretical efficiency is determined by the boiler temperature and the condenser temperature, so the boiler temperature should be made as high as possible relative to the furnace temperature, and the condenser temperature as low as possible (with room temperature the lowest practical limit). He estimates the “duty” of seven actual steam engines, with values from 8.6% to 57.5%.

Although from his work on the dependence of the freezing temperature on pressure Thomson is aware of the ideas embodied in the Clapeyron equation, he does not seem to derive that result. It must be kept in mind that Thomson was an extremely busy researcher in many areas. For example, in a personal letter, in 1850 he informed Stokes of what is now known as Stokes’s Theorem [57].

This concludes our history of thermodynamics prior to the acceptance of conservation of energy.

## 9. On Energy Conservation

Much of the following discussion is taken from secondary sources, especially Ref. [7]. For a more complete listing of those who, in the relevant time-period, came close to the mechanical equivalent of heat and/or energy conservation, see Kuhn’s article on energy conservation [23]. In many of these works, the author recognized that there is a mechanical equivalent of heat, but did not also recognize conservation of something involving a trade-off between heat and work.

**Mayer.** Mayer, a physician, in 1842 developed a theoretical argument for the mechanical equivalent of heat, based on the difference in specific heats at constant volume and at constant pressure. He attributed this difference to the work done at constant pressure as the volume expanded [58]. Perhaps without realizing it, he assumed, implicitly but correctly, that the internal energy of a real gas is basically independent of the gas interactions, and therefore is independent of the gas density. Without showing details of his calculation, he obtained a reasonable value for the mechanical equivalent of heat. Mayer performed no experiments himself, although he did employ specific heat data from Gay-Lussac [59]. Belatedly, but during his lifetime, Mayer’s work received the recognition it deserved [7]. Hutchinson discusses Mayer’s work, how it was confirmed by Joule’s 1844 work, and how Joule and Thomson’s further study of the effect of density on gas properties (the Joule-Thomson effect, 1854) helped establish the Kelvin scale [11].

On p.73 of the translation in Ref. [60], Mayer writes “we find the decrease in height of a mercury column compressing [a confined] gas equivalent to the quantity of heat associated with the compression. If we put the ratio of the specific heat capacities of the gas at constant pressure and constant volume respectively equal to 1.421, [logical gap to follow!] it turns out that the fall of a weight from a height of about 365 meters corresponds to the heating of an equal mass of water from 0 ${}^{\circ }$C to 1 ${}^{\circ }$C.”

Without telling us, in that logical gap Mayer has computed the mechanical equivalent of heat *a* and then used it to compute, from the known specific heat of water, the 365 m height of fall needed to raise the water temperature by 1 ${}^{\circ }$C. Employing $mgh=mc_{w}\Delta T$ with the specific heat of water $c_{w}$ in J/Kg$\cdot^{\circ }$C, we reverse the logic, and compute from Mayer’s information that $c_{w}=gh/\Delta T$ is about 3580 J/Kg$\cdot^{\circ }$C. Compared to the approximate modern value of 4200, this indicates that Mayer’s value for the mechanical equivalent of heat was a bit too small; rather than 4.19 J/Cal it was about 3.57 J/Cal. Not until his 1845 publication does Mayer indicate how he computes *a* and give his value. See p.84 of Ref. [60].

**Joule.** In late 1840, Joule published his observation of Joule heating, that wires heat up at a rate proportional to their electrical resistance and to the current squared [61,62]. Around 1842 Joule began a series of independent experiments that gave accurate values for the mechanical equivalent of heat. He first studied what is known as Joule heating due to electric current driven by chemical energy from a battery, and next studied what we now know as “Joule heating” due to electric current driven by electromagnetic induction from mechanical energy. In 1844, Joule wrote of some of his experiments on air, that “the heat evolved by compressing the air was found to be the equivalent of the mechanical power employed, and, vice versa, the heat absorbed in rarefaction was found to be the equivalent of the mechanical power developed.” As Hutchison notes, although in this paper Joule did not study the conversion of heat to work (all other things held constant), he did show that (all other things *not* held constant) work converts to heat and that (some) heat converts to work, in equivalent amounts [11]. In this same passage, Joule quotes Mayer as having in 1842 proposed such an equivalence, “without, however, attempting an experimental demonstration of its accuracy.” One cannot fail to recognize the methodical, thorough, and careful approach taken by Joule [63].

Joule next studied, in great quantitative detail, the direct production of heat due to mechanical energy. “He stirred a liquid, such as water or mercury, in a kind of churn driven by a falling weight. The range of descent of the weight enabled the work consumed to be exactly estimated, and a sensitive thermometer in the liquid measured the rise of temperature; thus the heat produced was accurately determined. ...The experiments proved that the work spent on the liquid and the heat produced were in direct proportion to one another” [64].

**Colding.** Colding, a Danish civil engineer, is largely ignored in textbook discussions of the history of energy conservation. Working at Oersted’s institute, he published in Scandinavian journals, which were largely unread out of the Scandinavian countries. In 1863, he wrote, in English, a summary of his work [65]. There he noted that in 1843 he gave many examples of heating due to compression of both liquids and solids, and developed an apparatus to study heating due to friction, in part to establish that the heating was proportional to the ‘mechanical power lost’. He published his results in 1847, with a value for the mechanical equivalent not far from that of Mayer.

**Holtzmann.** In 1845, Holtzmann (“a schoolteacher and competent mathematician” [66]), aware of work on the mechanical equivalence of heat but, apparently, not of energy conservation, considered Clapeyron’s extension of Carnot. Using the same method as Mayer he obtained a value for the mechanical equivalence of heat that substantially agreed with that of Joule. However, Holtzmann did not take the step of arguing that something is conserved, nor did he redefine the temperature scale via $T_{g}=a+t$, where Holtzmann took the temperature offset a=267 [7].

In his 1850 paper, discussed below, Clausius indicates that Holtzmann took $a^{\prime }$ as the work equivalent of a unit of heat [2]. (To avoid confusion with the temperature offset *a* we have appended a prime to Holtzmann’s *a*.) Clausius further indicates that Holtzmann gave the Carnot function as $C=p/(a^{\prime }\rho )$. Taking ρ=1/v and pv=R(267+t), and with $J^{-1}$ in place of $a^{\prime }$, this gives Clapeyron’s C=J[R(267+t)]. This is equivalent to taking $C∼1/T_{g}$.

**Helmholtz.** In 1847, Helmholtz wrote a paper mathematically enunciating energy conservation (he called it ‘force conservation’) [30]. First, he considered mechanical energy, including both what we would call kinetic energy and potential energy, showing that for central forces their sum is conserved. He then discussed heat, arguing that Joule’s many experiments show that heat and work are both quantities of the same type, what we would now call energy, and thus that total energy (for Helmholtz, ‘total force’) is conserved [30].

Helmholtz gave had specific objections to two proposals that treated frictional heating using the idea of a conserved heat fluid: (1) if friction involves thermal conduction and conserved heat, then friction would heat one surface and cool the other (rather than heat both, as is customarily experienced), and (2) if friction is produced by surface compression followed by abrasion (as in Rumford’s cannon experiments) then it would: (a) require improbably large compressions; (b) not apply to (unabradable) fluids; and (c) totally fail to explain the (nonabrasive) softening of iron by hammering. Helmholtz’s discussion thus supports the mechanical theory of heat.

## 10. Clausius 1850

In his foundational 1850 paper, Clausius builds on the theoretical work of Clapeyron (1834), and of Thomson (1849), but with the addition of energy conservation. Among his prefatory comments, Clausius argued, by considering steam, that latent heat is not merely hidden, but rather “has actually *no existence.*” He then distinguished between the “interior work” that is “necessary to overcome the mutual attraction of the particles”, and the “exterior work” (the usual dW) for the vapor “to procure room for itself, forcing back an outer pressure”. He wrote that the amount of heat required to change the state of a body depends on the process, and he referred “to that portion only of the *latent heat* which corresponds to the *exterior work.* The remainder of the latent heat and the entire amount of the sensible heat are independent of the manner in which the alteration is affected.” (Recall that “sensible heat” is what causes temperature change.) Clausius was struggling to deal with the issue of internal energy, which was to bother him for years. We now discuss his work more explicitly. Like Clapeyron, Clausius considers the ideal gas before steam.

### 10.1. Ideal Gas and Specific Heats

Clausius considers the air engine via a unit weight of ‘permanent gas’ (ideal gas), which satisfies Mariotte’s Law (Boyle’s Law) that pv is constant at fixed temperature *t*, and which also satisfies Gay-Lussac’s Law (Dalton’s Law) that, at fixed *v*, pressure changes vary linearly with temperature changes:

(50) pv=R(a+t).(1)

Here *t* is in Celsius; the offset a=267 used by Clapeyron, with more accurate measurements, has become a=273 for Clausius. Clausius notes that Regnault showed that there are small deviations from this law, decreasing as the gas density decreases. Clausius also defines the heat equivalent of work as *A* (the inverse of Holtzmann’s $a^{\prime }$) via

(51) $$\frac{\mathrm{heat}\;\mathrm{expended}}{\mathrm{work}\;\mathrm{produced}}=A.\;\left(4.\right)$$

He then analyzes the Carnot cycle between *t* and t−1, using a heat “function” Q(t,v) that he knows has meaning only for its differentials. His equation, when interpreted with the quantity *Q*, which despite appearances he knows is *not* a function of *t* and *v*, is

(52) $$\frac{d}{dt}\left(\frac{dQ}{dv}\right)-\frac{d}{dv}\left(\frac{dQ}{dt}\right)=\frac{AR}{v}.\;\left(II.\right)$$

This only *appears* to contradict the rules of calculus, since for a true function Q(t,v) the right-hand side must be zero. (This equation apparently confused some of his readers, since a later version of this work discusses the point more extensively.).

Solving the above equation, Clausius finds that

(53) $$dQ=dU+AR\frac{a+t}{v}dv,\;\left(IIa.\right)$$

where *U* is a true (path-independent) function of *t* and *v* (in units of heat, not units of energy). He has thus made a mathematical distinction associated with new physics. More generally, it corresponds to the modern

(54) dQ=dU+pdv=dU+dW.

Thus the thermodynamic energy function *U* appears, although not yet with units of energy.

He argues that “when a permanent [ideal] gas expands at a constant temperature, it absorbs only as much heat as is necessary to the exterior work produced by expansion”. That is, at fixed *t* he takes dU=0. Thus, more explicitly than Mayer, Clausius is aware that the internal energy of an ideal gas depends only on temperature. (Deviations from this were to be studied experimentally by Joule and Thomson in their porous plug experiments, where gas expands into a vacuum [67]). From (53) Clausius concludes that, at fixed temperature,

(55) $$\frac{dQ}{dv}=AR\frac{a+t}{v}.\;\left(9.\right)$$

Clausius further argues that the function *U* does not depend on *v*, so that, with $c_{v}$ (he uses *c*) a function only of *t*, (53) becomes

(56) $$dQ=c_{v}dt+AR\frac{a+t}{v}dv.\;\left(IIb.\right)$$

He comments “and it is even probable that the quantity *c*, which denotes the specific heat of the gas at constant volume, is itself a constant.” He then writes, at fixed volume, that by (55) the specific heat per weight is

(57) $$\frac{dQ}{dt}=c_{v}.\;\left(10.\right)\;[\mathrm{fixed}\;\mathrm{volume}]$$

Clausius now turns to the specific heat at fixed pressure $c_{p}$, which he calls $c^{\prime }$ [68]. Since at fixed *p* the ideal gas satisfies pdv=Rd(a+t)=Rdt, he then writes that, at fixed pressure, the specific heat per weight is

(58) $$\frac{dQ}{dt}=c_{p}=c_{v}+AR.\;\left(10a.\right)\;[\mathrm{fixed}\;\mathrm{pressure}]$$

Clapeyron’s form yields this if $C=a+t=T_{g}$.

Clausius goes on to define specific heats $\gamma ,\gamma^{\prime }$ per volume, as in common use then. Defining the constant $k\equiv c^{\prime }/c$, he shows that it is inversely proportional to γ, and comments that it agrees with experiments by Dulong.

### 10.2. Ideal Gas and Adiabatic Condition

Clausius now considers processes without heat flow (adiabatic), so dQ=0. Then, by (53),

(59) $$cdt+AR\frac{a+t}{v}dv=0,\;\left(13.\right)$$

which leads to

(60) $$v\frac{AR}{c}\left(a+t\right)=const;$$

or, with $AR/c=c^{\prime }/c-1=k-1$,

(61) $$v^{k-1}\left(a+t\right)=const.$$

From this he shows that the heat absorbed from a reservoir at fixed *t* satisfies

(62) $$\frac{p}{p_{0}}={\left(\frac{v_{0}}{v}\right)}^{k},\;\left(16.\right)$$

and notes agreement with Poisson, who had built on Laplace [7,46]. However, this fundamental result, the adiabatic condition, had earlier been established only using a faulty theory based on conservation of heat; whereas Clausius has established it using a correct theory based on conservation of energy. It is one of the foundations of thermodynamics.

Considering that *t* is constant, Clausius notes that

(63) $$dQ=AR\frac{a+t}{v}dv;\;\left(17.\right)$$

integration gives

(64) $$Q-Q_{0}=AR\left(a+t\right)\log\frac{v}{v_{0}}.\;\left(18.\right)$$

This is to be compared with Clapeyron’s 3.21 (21).

### 10.3. Thermodynamic Efficiency

Returning to an infinitesimal Carnot cycle for an ideal gas, Clausius considers expansion and compression dv and reservoir temperature difference dt. The heat entering at the hot reservoir is dQ=(dQ/dv)dv. The net pressure change on going from one reservoir to the other is dp=(R/v)dv. Hence the work done in a cycle is dW=dpdv=(R/v)dvdt, so the thermodynamic efficiency is

(65) $$\frac{dW}{dQ}=\frac{R}{v(dQ/dv)}dt.$$

However, by Clapeyron’s definition of *C* from (17), we also have dW/dQ=dt/C. Hence Clausius obtains

(66) $$\frac{dQ}{dv}=\frac{RC}{v}.\;\left(IV.\right)$$

Comparison with (55) gives

(67) C=A(a+t),

although Clausius expresses this important result only in words. Clausius has thus derived, with $T_{g}=a+t$, the equivalent of $C=AT_{g}$.

### 10.4. Steam

Expanding on Clapeyron, Clausius also develops the theory for fluid-vapor mixtures (e.g., water-steam). At common vapor and fluid temperature *t*, and at its “maximum density” (likely this meant saturation) he defines:

(1) s,σ as the volumes of a unit mass of vapor (steam) and of fluid (water);
(2) m,μ as the masses of vapor and fluid;
(3) h,c as the specific heats per mass of vapor and fluid.
(4) *r* as the latent heat per vapor mass (rather than Clapeyron’s latent heat per vapor volume *k*).

Then for temperature increase dt between thermal reservoirs, so dp=(dp/dt)dt, and vapor mass change dm (with corresponding negative fluid mass change) for each reservoir, so dv=(s−σ)dm, he has

(68) $$\mathrm{work}\;\mathrm{produced}=\left(s-\sigma \right)\frac{dp}{dt}dm\;dt.\;\left(5.\right)$$

This is to be compared with what in (26) we have called Clapeyron’s [4.2].

Accounting for the liquid-vapor specific heat difference c−h and the temperature dependence of the latent heat *r* (both per mass) he has

(69) $$\mathrm{heat}\;\mathrm{expended}=\left(\frac{dr}{dt}+c-h\right)dm\;dt.\;\left(7.\right)$$

Equating work times *A* (the heat equivalent of work) to the net heat, which corresponds to no change in the energy of the system, Clausius obtains

(70) $$\frac{dr}{dt}+c-h=A\left(s-\sigma \right)\frac{dp}{dt}.\;\left(III.\right)$$

We do not show details of this complex Carnot cycle, with mass changes $dm,dm^{\prime }$ in the isothermal legs and $\delta m,\delta m^{\prime }$ in each of the adiabatic legs. Clausius had the precedent of Thomson’s earlier work on the four legs of an ideal gas Carnot cycle. Again, as for the ideal gas in the discussion above Equation (42), it is not necessary to obtain all four unknowns. This is one of the few places where Clausius may have a debt only to Thomson, rather than also to Clapeyron.

### 10.5. Steam and Clausius-Clapeyron Equation

Part II of Clausius’s Memoir applies Carnot’s efficiency definition to steam. He has already applied it to the ideal gas, deriving (67). Recall that, unlike the requirement of no net energy change on going around a Carnot cycle, the Carnot efficiency involves only the heat provided by the higher temperature, not the net heat. In an unnumbered equation, Clausius writes this as

(71) $$\left[dQ=\right]rdm.$$

With dQ from (71), dW from (68), and dW/dQ=dt/C from Clapeyron, he obtains

(72) $$r=C\left(s-\sigma \right)\frac{dp}{dt},\;\left(V.\right)$$

which is a form of the Clausius-Clapeyron equation; compare to (28). Clausius notes that Clapeyron has already derived Clausius’s (IV.) and (V.).

Clausius goes on to analyze data on air and on steam. For air his Table I shows that the *C* obtained by Clapeyron, Thomson, and himself are consistent at the four temperatures he considers.

For steam, he combines (70), (72) and (67) to obtain

(73) $$\frac{dr}{dt}+c-h=\frac{r}{a+t}.\;\left(32\right)$$

His Table VI shows that for $0,50,100,150,200{\;}^{\circ }$C the quantity *h* is negative, a seemingly extraordinary result that was simultaneously noted by Rankine [69], although later Clausius questioned Rankine’s derivation [70]. Unlike Thomson, Clausius does not assume that vapor pressure is proportional to density.

Certainly, for thermodynamic stability the overall specific heat of the system cannot be negative. Ref. [71] argues that the actual specific heat of each component differs from the specific heat at constant volume, or $c_{v}$, and gives the corrections. If the steam and water are in equilibrium with one another, one should calculate the specific heats with each having the same pressure and chemical potential. However, the pressure and chemical potential vary on heating, as do the densities of each phase. The saturated vapor-fluid system is indeed complex.

With this proviso about the meaning of *c* and *h*, if in (73) *c* nearly equals its value for bulk water, and if *r* is so large that r/(a+t) dominates dr/dt and *c*, then *h* must be negative. Consistent with this, in the data cited above, as *t* increases, so *r* decreases and *h* becomes less negative. Hutchison notes that the operation of the cloud chamber depends upon this property [11].

## 11. Thomson 1851

As noted at the outset of this article, Thomson’s 1851 work introduces “thermo-dynamics” as a noun [3]. Thomson calls energy conservation the first law of thermodynamics. He calls the statement, loosely speaking, that heat cannot be converted to work (all other things held constant) the second law of thermodynamics. Thus he replaces Clausius’s heat-flow-based version of the second law, loosely speaking, that heat cannot flow from cold to hot (all other things held constant). He shows that the two statements of the second law are equivalent. This work had five parts published in 1851; we discuss only the first four parts. Part VI, on thermo-electric currents, was published in 1854 and Part VII, on thermo-elastic, thermomagnetic, and pyroelectric phenomena, in 1856; we discuss only part VI for what it says about combinations of reversible Carnot cycles.

### 11.1. Part I. Fundamental Principles

For a volume change dv at fixed pressure *p*, Thomson writes for the work done

(74) $$\left[dW=\right]pdv,\;\left[I.1\right]$$

He also writes that the heat entering is

(75) $$\left[dQ=\right]Mdv+Ndt,\;\left[I.2\right]$$

Note that $N\equiv c_{v}$.

Although Thomson has read Clausius, and Clausius has referred to Holtzmann’s mechanical equivalent of heat $a^{\prime }$, Thomson defines the mechanical equivalent of heat to be *J* (presumably an homage to his friend Joule). Perhaps Thomson has in mind other uses of *a*, such as the temperature offset in going from Celsius to the absolute ideal gas temperature $T_{g}$. Thomson also does not note that *J* relates to Clausius’s heat equivalent of work *A* by $J=A^{-1}$.

Thomson then writes that “the mechanical measure of the total external effect” is

(76) $$\left[dW-JdQ=\right]\left(p-JM\right)dv-JNdt,$$

Recognizing that this is the differential of a true function of *v* and *t*, he notes that the order of the derivatives should not matter (as in Maxwell relations), so that

(77) $$\frac{d(p-JM)}{dt}=\frac{\left(-JN\right)}{dv},\;\left(1\right)$$

or

(78) $$\frac{dp}{dt}=J\left(\frac{dM}{dt}-\frac{dN}{dv}\right).\;\left(2\right)$$

These derivatives are, of course, partial derivatives, either at fixed *v* or at fixed *t*.

Thomson then considers, for a reversible Carnot cycle with small volume change dv of each reservoir and small temperature difference τ between the reservoirs, that the total work done is dW=dpdv=(dp/dt)dtdv=(dp/dt)τdv. He argues that, when in contact with the hot reservoir, on expansion by dv at dt=0, that (75) gives heat input dQ=Mdv. Defining the quantity μ (he calls this “Carnot’s function”; it is inversely proportional to the efficiency) as in his 1849 paper, with units of *J* times inverse temperature, he obtains

(79) $$\mu \equiv \frac{1}{\tau }\frac{dW}{dQ}=\frac{1}{\tau }\frac{\frac{dp}{dt}\tau \;dv}{M\;dv}=\frac{1}{M}\frac{dp}{dt},\;\left(4\right)$$

where μ is a function only of *t*.

Paragraph 22 comments that the Carnot efficiency gives only a “fraction of the whole mechanical equivalent of the heat supplied; the remainder being irrecoverably lost to man, and therefore ‘wasted,’ although not *annihilated.*” He is not fully reconciled with the fact that a reversible Carnot engine is the best that can be done to convert heat to work.

### 11.2. Part II. Finite Temperature Differences

Now Thomson turns to reversible Carnot cycles, with reservoir temperatures (in Celsius) for the upper (heater) reservoir of *S* and for the lower reservoir (refrigerator) of *T*. He takes heat flow *H* entering the working medium from hot reservoir *S* and heat flow *R* leaving to cold reservoir *T*. To develop the theory for finite temperature differences, he first considers that q+dq enters from the hot reservoir at *t* and *q* leaves to the cold reservoir at t−dt. By what he has called the first Law the net work is dW=Jdq. From the definition μ in (79) and with heat *q* corresponding to dQ, one has Jdq=qμdt. On integrating from *T* to *S* this gives

(80) $$\log\frac{H}{R}=\frac{1}{J}\int_{T}^{S}\mu \;dt,\;\left(5\right)$$

so

(81) $$R=H\exp\left[-\frac{1}{J}\int_{T}^{S}\mu \;dt\right].\;\left(6\right)$$

By energy conservation

(82) W=J(H−R).(7)

A lengthy discussion based on steam engine data follows.

Near the end of Part II, Thomson turns to a result “suggested to me by Mr. Joule” in a letter dated 9 December 1848, that μ might be “inversely as the temperatures from zero.” Thomson then writes

(83) $$\mu =J\frac{E}{1+Et}.\;\left(11\right)$$

Although actually derived by Clausius, rather than merely being suggested by Joule, Thomson writes that this equation “is also adopted by Clausius.” This point is to return in a published exchange of letters between Clausius and Thomson in 1856.

Apparently Thomson did not recall his earlier result (47), for an ideal gas isotherm, which can be re-written as μ=(W/Q)E/(1+Et). Had he set W=JQ, which he accepted in 1851 but not in 1849, he would now have derived $\mu =J/T_{g}$. Although Joule’s prescience must be commended, Thomson’s wording denies to Clausius his full due.

From (83) it follows that

(84) $$\int_{T}^{S}\mu \;dt=J\log\frac{1+ES}{1+ET},$$

which by (81) yields

(85) $$\frac{R}{H}=\frac{\frac{1}{E}+T}{\frac{1}{E}+S}.\;\left(13\right)$$

(Thomson implicitly sets H=1.) Then, by (82),

(86) $$W=J\frac{S-T}{\frac{1}{E}+S}.\;\left(12\right)$$

With ideal gas temperatures $T_{R}=T+E^{-1}$ and $T_{H}=S+E^{-1}$, (85) becomes

(87) $$\frac{R}{T_{R}}=\frac{H}{T_{H}},$$

which is equivalent to the reversibility condition (4) when signs for heat entering and leaving are appropriately defined. However, Thomson does not attribute any special significance to (85).

### 11.3. Part III. Applications to Physical Properties of all Substances

Thomson next derives, for any material, the difference $c_{p}-c_{v}$, using his relations (2) and (3). He re-arranges his (2), and uses his (4), to obtain

(88) $$\frac{dN}{dv}=\frac{d}{dt}\left(\frac{1}{\mu }\frac{dp}{dt}\right)-\frac{1}{J}\frac{dp}{dt}.\;\left(14\right)$$

Starting from (75), at constant pressure, so

(89) $$0=\frac{dp}{dv}dv+\frac{dp}{dt}dt,$$

he then notes that the specific heat at constant pressure $K\left[=c_{p}\right]$ satisfies

(90) Kdt=Mdv+Ndt.

Eliminating dt and dv from these equations yields

(91) $$M=\frac{dt}{dv}\left(K-N\right)=\frac{-\frac{dp}{dv}}{\frac{dp}{dt}}\left(K-N\right).\;\left(15\right)$$

Use of his (4), or dp/dt=μM gives the general form

(92) $$\left[c_{p}-c_{v}=\right]K-N=\frac{{\left(\frac{dp}{dt}\right)}^{2}}{-\mu \frac{dp}{dv}},\;\left(16\right)$$

where leftmost is the conventional specific heat notation.

Introducing the reciprocal of the compressibility κ and the coefficient of thermal expansion *e* (notation alert!) he defines

(93) $$\kappa =v\left(-\frac{dp}{dv}\right),\;e=\frac{1}{\kappa }\frac{dp}{dt}.\;\left(17\right)$$

Thomson then rewrites his (14), (16), and (3) as

(94) $$\frac{dN}{dv}=\frac{d\left(\frac{\kappa e}{\mu }\right)}{dt}-\frac{\kappa \;e}{J},\;\left(18\right)$$

(95) $$K-N=v\frac{\kappa \;e^{2}}{\mu },\;\left(19\right)$$

(96) $$M=\frac{1}{\mu }\kappa \;e.\;\left(20\right)$$

With $\mu \to T^{-1}$, κ→K, and e→β, Equation (95) is equivalent to the result

(97) $$c_{p}-c_{v}=TvK\beta^{2}$$

traditionally taught in thermodynamics courses. (Here work and heat take the same units, so J→1).

Thomson now defines, for dQ=0 in (76), the temperature increase θ (notation alert relative to his *temperature*θ!) due to a volume change dv<0

(98) $$\theta =-\frac{dt}{dv}dv=\frac{M}{N}dv=\frac{1}{N}\frac{\kappa \;e}{\mu }dv=\frac{\kappa \;e}{\mu K-v\kappa \;e^{2}}dv.\;\left(21\right)$$

He notes the implication that if a substance contracts on heating (as with water near the freezing temperature), then its temperature decreases on compression. Recall that Clapeyron made a similar comment, with pressure increase in place of volume decrease.

After showing that the general results reduce to his previously obtained results for the ideal gas, Thomson applies his results to steam.

### 11.4. Part IV. Measurement of, for a Compressed Fluid, the Mechanical Work and the Heat Produced

This is known as the Joule-Thomson effect. We will not consider this part because we believe they needed a better theory, such as that of Van der Waals, to interpret deviations from the ideal gas law. However, more importantly we believe that despite their efforts to establish an accurate practical temperature scale, ultimately it was Clausius who correctly showed that the inverse of the absolute ideal gas temperature $1/T_{g}$ is the integration factor for entropy, and therefore that the thermodynamic temperature $T=T_{g}$. Nevertheless, the Joule-Thomson effect, interpreted using the Van der Waals equation or a more accurate variant, does yield corrections to the ideal gas law temperature [67]. See Section 13.

### 11.5. Part VI. Thermo-Electric Currents

This work dates to 1854. Paragraph 101 contains the reversibility condition (with *Q* for Thomson’s *H*) as

(99) $$\sum_{i}\frac{Q_{i}}{T_{i}}=0.$$

Thomson comments that this follows from the Second Law [72]. In a later publication, he notes that, for Joule’s suggested μ=JE/(1+Et), Equation (99) is equivalent to an earlier result, presumably his (85), for a complex cycle built from individual Carnot cycles [73].

It is important to recognize that, unlike Clausius later that same year, Thomson did not write (99) in integral form. Had he done so, he might have derived the equivalent of what Clausius did later that year; namely, that for reversible processes $\oint dQ/T_{g}=0$ for any path in *p*-*v* space, which implies a thermodynamic state function (entropy) associated with temperature. This was not beyond Thomson’s very significant mathematical capabilities; as already noted, in an 1850 letter to Stokes he revealed a few mathematical results, including what is now known as Stokes’s Theorem. This latter states the condition for a state function *S* to exist, and is equivalent to $\partial_{v}\partial_{p}S=\partial_{p}\partial_{v}S$.

## 12. Clausius 1854

First Clausius recapitulates his earlier work, but in more generality than for the ideal gas, so that on the right-hand-side of (52) he replaces R/v by dp/dt

(100) $$\frac{d}{dt}\left(\frac{dQ}{dv}\right)-\frac{d}{dv}\left(\frac{dQ}{dt}\right)=A\frac{dp}{dt}.\;\left(3\right)$$

He then argues that heat flow *Q* goes into interior work and exterior work; where interior work does not change in a reversible cyclic process [74].

### 12.1. Clausius’s Two Types of “Equivalences of Transformation”

Clausius’s next step brings him very close to the concept of entropy, which he does not fully formulate or name until 1865. He introduces the idea of *equivalences of transformation* (of heat into work) and two types of “equivalence values”. Initially they are both unspecified functions; it is his goal to determine them. What follows employs his definitions but somewhat modifies the reasoning in his paper. His later use of *entropy* is a Greek echo of *equivalence,* and led him to the use of *S* rather than, in his 1854 paper, the symbol *N*.

(1) One type, the equivalence value function f(t), applies when work *W* is completely converted into unit heat Q=1 at a reservoir of temperature *t*, with f(t)>0. For more general *Q*, f(t) appears in the form Qf(t). Conversely, if heat *Q* at *t* completely converts to work, then its equivalence value is −Qf(t):
  (101) equivalencevalue−Qf(t)ifQattconvertstoW
(2) The other type, the equivalence value function $F(t_{1},t_{2})$, applies when unit heat $\bar{Q}=1$ spontaneously flows from reservoir $t_{1}$ to reservoir $t_{2}$, as occurs if $t_{1}>t_{2}$, with $F(t_{1},t_{2})>0$. For more general $\bar{Q}$, $F(t_{1},t_{2})$ appears in the form $\bar{Q}F\left(t_{1},t_{2}\right)$:
  (102) $$\mathrm{equivalence}\;\mathrm{value}\;\bar{Q}F\left(t_{1},t_{2}\right)\;\mathrm{if}\;\bar{Q}\;\mathrm{flows}\;\mathrm{from}\;t_{1}\;\mathrm{to}\;t_{2}$$

A Carnot cycle involves both processes. However, direct thermal contact, which produces no work, involves only the second. For $\bar{Q}>0$, this occurs only for $t_{1}>t_{2}$. Clausius first relates *F* to *f*, and then finds *f*.

He first notes that, by reversing the process involving $t_{1}$ and $t_{2}$, the sign of *F* should reverse, so

(103) $$F\left(t_{2},t_{1}\right)=-F\left(t_{1},t_{2}\right).\;\left(4\right)$$

He next gives a figure showing a complex reversible Carnot cycle in *p*-*v* space, with $t>t_{1}>t_{2}$. This introduces K${}_{1}$ and K${}_{2}$ as intermediary reservoirs, but in a second process their presence will cancel.

He lets heat at *t* convert completely to work *W* (Q→W) and has heat $Q_{1}$ go from $t_{1}$ to $t_{2}$. He now imposes the condition that for real reversible processes one must have no net change in the equivalence value of a combination cycle involving the three reservoirs *t*, $t_{1}$, and $t_{2}$. Therefore

(104) $$-Qf\left(t\right)+Q_{1}F\left(t_{1},t_{2}\right)=0;\;\left(5\right)\;(\mathrm{produce}\;\mathrm{work})$$

Thus, if f(t) and $F(t_{1},t_{2})$ are known, then on specifying *Q* we obtain $Q_{1}$.

He now considers a reversed process with $W\to Q^{\prime }$ to a different reservoir $t^{\prime }$, and with $Q_{1}$ reverse driven from $t_{2}$ and $t_{1}$. Again taking zero net change in the equivalence value yields

(105) $$Q^{\prime }f\left(t^{\prime }\right)+Q_{1}F\left(t_{2},t_{1}\right)=0.\;\left(6\right)\;(\mathrm{receive}\;\mathrm{work})$$

Addition of (104) and (105), and the anti-symmetry property of *F*, then gives

(106) $$-Qf\left(t\right)+Q^{\prime }f\left(t^{\prime }\right)=0.\;\left(7\right)\;(\mathrm{no}\;\mathrm{net}\;\mathrm{work})$$

The net effect is that *Q* is lost by reservoir K, and $Q^{\prime }$ is gained by reservoir K’; the intermediary reservoirs K${}_{1}$ and K${}_{2}$ are unaffected, and no work is done.

I now paraphrase Clausius’s reasoning [75]. He argues, in effect, that one can produce the usual simple Carnot cycle with *Q* going from $t^{\prime }$ to *t*, provided that not all of the heat *Q* goes to *t*, but rather $Q-Q^{\prime }$ of that heat becomes work *W*. *This step implicitly uses energy conservation.* Using (104), this Carnot cycle has equivalence value

(107) $$-\left(Q-Q^{\prime }\right)f\left(t^{\prime }\right)+QF\left(t^{\prime },t\right)=0.$$

Clausius now notes that (106) relates $Q^{\prime }$ to *Q*; when substituted into (107) and *Q* is factored out, one obtains

(108) $$F\left(t,t^{\prime }\right)=f\left(t\right)-f\left(t^{\prime }\right).\;\left(8\right)$$

Thus there now is only one unknown function f(t). At this point Clausius rewrites *f* in terms of a new but still unknown function with a rather suggestive name:

(109) $$f\left(t\right)=\frac{1}{T}.\;\left(9\right)$$

where T(t) is a new, material-independent function of temperature *t* that he proceeds to determine.

### 12.2. The Reversibility Condition: An Early Version of Entropy

Clausius rewrites (106) to permit arbitrary signs for the heat, and then defines its sum for all transformations *N*, which for reversible transformations is zero:

(110) $$N=\sum_{i}\frac{Q_{i}}{T_{i}}=0.\;\left(10\right)$$

He now takes the crucial step of considering the continuous case, defining

(111) $$N=\oint \frac{dQ}{T}\;\left(11\right)$$

and notes that on going around any path, for reversible transformations

(112) $$N=\oint \frac{dQ}{T}=0.\;\left(II\right)$$

Clausius has thus obtained the equivalent of entropy *S*; however, not until 1865 did he arrive at the name ‘entropy’ and recognize its non-decreasing nature. Properly, however, Carnot cycles have zigs and zags that do not disappear in the limit of infinitesimal cycles, so without further consideration the integral form ∮dQ/T is problematic. However, a single infinitesimal Carnot zigzag labeled ABC (one adiabat and one isotherm) can be replaced by a single infinitesimal straight line AC by using energy conservation. This permits smoothing the zigzags in the limit of infinitesimal cycles [18].

### 12.3. Finding the Absolute Temperature

Having written his first equivalence function in terms of the unknown function T(t), Clausius proceeds to use the reversibility condition to determine that function. Thus, the condition that entropy exists is also the condition that determines the temperature *T*, up to an arbitrary scale factor [18].

He now notes that, because of (112), dQ/T is a perfect differential, so

(113) $$\frac{d}{dt}\left(\frac{1}{T}\frac{dQ}{dv}\right)=\frac{d}{dv}\left(\frac{1}{T}\frac{dQ}{dt}\right).$$

Using T=T(t), the right-hand-side has only one term. With a single un-shown step of algebra, (113) leads to

(114) $$\frac{dQ}{dv}\frac{dT}{dt}=T\left[\frac{d}{dt}\left(\frac{dQ}{dv}\right)-\frac{d}{dv}\left(\frac{dQ}{dt}\right)\right].$$

Comparison to (100) then leads to

(115) $$\frac{dQ}{dv}\frac{dT}{dt}=AT\frac{dp}{dt}.$$

Because T=T(t) we have dp/dt=(dp/dT)(dT/dt), so that (115) can be written as

(116) $$\frac{dQ}{dv}=AT\frac{dp}{dT}.\;\left(13a\right)$$

Although Clapeyron’s 1834 theory was based on heat conservation, as noted earlier, certain of Clapeyron’s results were valid. One of these is Clapeyron’s Equation (18), given above, or dQ/dv=Cdp/dt, to which Clausius now refers. Clausius concludes that Clapeyron’s unknown *C* relates to his own still-unknown *T* by C=AT, which he does not bother to write down. However, he had already derived the equivalent of $T=T_{g}$ in 1850: see Equation (67). So this is his second derivation of C=AT.

Clausius now turns to the task of finding T(t) by considering the ideal gas. For the ideal gas the total energy does not depend on *v*, so dQ=AdW. Then the left-hand-side of (116) becomes

(117) $$\frac{dQ}{dv}=A\frac{dW}{dv}=Ap=AR\frac{a+t}{v}.$$

Further, from dT=(dT/dt)dt and the ideal gas law the right-hand-side of (116) becomes

(118) $$\frac{dp}{dT}=\frac{R}{v}\frac{dt}{dT}.$$

Then (116) can be rewritten as

(119) $$AR\frac{a+t}{v}=AT\cdot \frac{R}{v}\frac{dt}{dT},\;\mathrm{or}\;\frac{dT}{T}=\frac{dt}{a+t}.$$

Thus, up to a scale factor taken to be unity, Clausius finds the absolute temperature

(120) T=a+t.

This *t* is in Celsius, so *T* is the ideal gas scale $T_{g}$, which is material-independent and, by (110), takes on an absolute meaning (up to a scale factor). We stress that although Thomson proposed this relationship, he did not derive it, unlike Clausius.

To summarize, from Carnot’s ideas and conservation of energy, Clausius determined a material- independent (universal) and absolute temperature scale, and (up to an unknown constant, later provided by Nernst in the Third Law of Thermodynamics) a new extensive variable, the entropy. It is extraordinary how such abstract ideas can yield such a precise theoretical framework. Recall that entropy is uniquely defined up to a scale factor, and from entropy and energy temperature acquires a unique definition. Thomson’s initial view that the Carnot efficiency uniquely defines temperature was mistaken. Clausius derived a condition that yields both entropy and temperature.

As noted, in 1850 Clausius had already derived $C=AT_{g}$. In 1851, Thomson, using Joule’s equivalent suggestion $\mu =J/T_{g}$ (equivalent to Clausius’s derived result), obtained the reversibility condition. This shows that Clausius did not have to go through his equivalence value argument at all to obtain the reversibility condition for a single Carnot cycle. Moreover, although by mid-1854 Thomson had generalized to an infinite sum over Carnot cycles, only Clausius took the crucial step of generalizing the sum over an infinite number of Carnot cycles into a closed path integral.

## 13. Thomson-Joule 1854

Joule and Thomson studied the effect of density on gas properties by measuring the temperature of a gas that freely expanded into a vacuum (their porous plug experiment) [67]. They found that the gas cooled slightly because the expanded state has less (negative) binding energy than the compressed state; to compensate it must also have less kinetic energy, meaning that it cools. The pressure-decreasing interaction term in the later van der Waals equation shows that the effect goes as the square of the density; however, Joule and Thomson did not have the benefit of this theory.

In a lengthy article in the Transactions of the Royal Society, June 1854, Joule and Kelvin proposed a temperature scale *t* based on what Thomson calls Carnot’s function μ [67]. They took

(121) $$t=\frac{J}{\mu }.\;\left(6\right)$$

Thus *t* is the ideal gas scale $T_{g}$. The theoretical part of the paper is contained in Section 5 “Physical Properties of Air expressed according to the Absolute Thermodynamic scale of Temperature”. This article basically repeats previous work deriving μ. Of historical interest, it is extensively discussed in Ref. [76].

We do not further discuss this or related work because we have earlier argued that this approach to determining an appropriate temperature scale cannot give a unique scale, whereas the approach of Clausius that yields entropy yields a unique scale up to a constant factor. Ref. [11] concludes by discussing Thomson’s change of view of the temperature scale, following the Joule-Thomson experiments.

## 14. Priority Disputes

1856 saw an exchange of two published letters between Clausius and Thomson [73,77]. Clausius comments on Thomson’s citation of Joule’s unpublished form for μ(T) using $T_{g}$, noting that a few months earlier that year Thomson referred to it as “Mr. Joule’s conjecture”. Clausius wrote that Thomson ascribed to Joule “the theorem”, presumably referring to what Clausius himself had derived; in 1851 Thomson wrote “suggested to me by Mr. Joule”. Further, Clausius notes that Holtzmann in 1845 had derived that same result using *heat* conservation, and that he (Clausius) was the first to derive “that theorem” using *energy* conservation [73]. Clausius seemed more concerned with Holtzmann’s priority over Joule for stating the result first than his own priority for giving the first well-founded derivation.

Thomson responded in the following letter, summarizing his previous statement in more detail than given by Clausius [77]. There Thomson notes that “the heat evolved in terms of the work spent in the compression of a gas” (at constant temperature) “is exactly the mechanical equivalent of the former.” Thomson calls this “Mayer’s hypothesis”, and indicates that he had called it that earlier in 1856. He further notes that it “is also assumed by Clausius without any reason from experiment.” However, in 1850 Clausius directly invoked a well-known experimental fact, in the paragraph before his Equation (9), that [2]:

“Gases exhibit in their deportment, particularly as regards the relations of volume, temperature and pressure, expressed by the laws of M.[ariotte] and G.[ay-Lussac], so much regularity as to lead us to the notion that the mutual attraction of the particles which takes place in solid and fluid bodies is in their case annulled.”

Nevertheless, Thomson goes on to note that “Mayer’s hypothesis’ was “at least approximately, verified by his [Joule’s] experiments” up to December of 1848, and goes on to cite the Joule-Thomson porous-plug experiments as having shown Joule’s conjecture “to be not rigorously, but very approximately true” [77].

- I think it fair to say that Mayer is the first to publish the idea of energy conservation and to obtain a value for the mechanical equivalent of heat. However, without further experimental support, as in the independent and extensive and careful work of Joule, it would not have served as a foundation for others to build upon. On the other hand, the underlying implication, not stated by Mayer, is that the constituents of the gases are so weakly interacting that volume changes due to expansion or compression do not affect their interactions.
- Thomson expended great effort in the laboratory to test his friend Joule’s “conjecture” that $\mu =J/T_{g}$. But Thomson strategically labeled and downgraded Mayer’s more well-founded but implicit assumption, that the constituents of an ideal gas interact only weakly, to the level of a “hypothesis”. In my opinion, this is a loss to the physics community. Mayer’s unstated but well-founded hypothesis about the lack of interactions in the ideal gas should be taught at the outset of *all* thermodynamics courses, in part because it so readily yields a value for the mechanical equivalent of heat. Further, as argued above, the relationship $\mu =J/T_{g}$ did not define temperature uniquely; uniqueness was first shown to follow from the integration factor argument of Clausius. Modern arguments, depending on the analysis of a Carnot cycle, and its generalization to arbitrary reversible cycles, are simpler than the argument of Clausius.
- S. Thompson, in his biography of Kelvin, condemns Clausius as arriving at a “vague statement” that heat tends to pass from hot to cold [52]. However, a similar comment can also be made about Kelvin’s initial statement of his version of the Second Law of thermodynamics. Moreover, Thompson also seems to appreciate neither the origin of Thomson’s specification of the reversibility condition in a “conjecture” rather than a theoretical deduction, nor Clausius’s extension of it *in integral form* to develop the entropy [78].
- Although I have read comments attributing to nationalism the priority jockeying between Thomson, Tait and Joule, on the one hand, and Clausius and Mayer on the other hand, I think it is more likely that it was a matter of personal loyalty in the staking of claims. To my knowledge, of all of the participants during the development of thermodynamics, only Thomson and Joule were friends. On the contrary, in 1862 the English Tyndall supported the German Clausius, likely simply out of a sense of fairness; after this Kelvin’s friend Tait entered the fray. I believe that Thomson had it within his ability to call off the polemical Tait; if Thomson had done so, various disagreements would have been more amicable, but perhaps in that case neither Joule nor Thomson would have had their eponymous units of energy and temperature. Not until somewhat later did Thomson and the German Helmholtz (a distant relative of the American William Penn) become good friends.
- By 1860 Maxwell, and by 1873 Gibbs, had entered the picture. These two had a calming influence. Gibbs wrote:
  “in the memoir of Clausius ...the science of thermodynamics came into existence”. However, he goes on with “In the development of the various consequences of the fundamental propositions of thermodynamics, as applied to all kinds of physical phenomena, Clausius was rivaled, perhaps surpassed, in activity and versatility by Sir William Thomson” [78]. Here Gibbs implicitly refers to Thomson’s far-sighted applications of thermodynamics to elasticity and to thermoelectricity.

As I hope this article shows, Clausius’s work is still not fully appreciated as forming the basis for both entropy and temperature. The unit of temperature is indeed the Kelvin, and to Kelvin we owe a great debt for early thermometry. However, it was Clausius who rigorously established that the ideal gas, or Kelvin scale, is the unique thermodynamic temperature.

## 15. Clausius and Entropy 1865

Although in the late 1850s Clausius did fundamental work in the kinetic theory of gases by developing the concept of the mean-free-path, thermodynamics still occupied his mind. In 1862 he introduced the term “disgregation” [79], a measure of the separation of the molecules of an object, with the same units as entropy. Disgregation has not turned out to be a fertile concept, but it was a stepping stone on Clausius’s path to entropy. For more on disgregation see Klein’s article on Gibbs’s view of Clausius [80], and Darrigol’s article on Gibbs’s mixing paradox [81].

On the other hand, Clausius’s 1865 introduction of “entropy” (from a Greek root for transformation) and his explanation in terms of reversible and irreversible processes, has become part of the terminology of daily life [38].

Although for a time Clausius’s terminology of entropy, disgregation, ergal, and virial “were for Maxwell the butt of jocular barbs” [82], all were well-defined (if not all useful) terms; ‘entropy’ and ‘virial’ have been adopted into general physics usage, but ‘ergal’ lost out to ‘potential energy’, and ‘disgregation’, with entropy units, just disappeared. Initially Maxwell was misled by Tait’s misunderstanding of ‘entropy’, but eventually Maxwell corrected himself and brought that to Tait’s attention.

## 16. Rankine

For my knowledge of the contributions of Rankine, works by Hutchinson and Marsden were extremely valuable [83,84,85]. These confirmed my own independent conclusion from a desultory reading of Rankine’s works, that Rankine, an engineer motivated by the desire to improve the steam engine, had a strong sense of what was needed to develop what we now call thermodynamics, but little of what he produced was unassailably correct. Sometimes his physics was correct and sometimes his new terminology stuck, but nearly as often his physics was wrong (his vortex model of an atom [84]) and his terminology did not stick (“metabatic” function, “metabaric” function, “actual energy”). I conclude that, despite his dissemination of useful methods to the engineering community, in terms of rock-solid physics he served more as a cheerleader and encourager for William Thomson (Kelvin) and Clausius, who made the major contributions [83,84,85]. Maxwell could not be persuaded to contribute something for Rankine’s obituary [85], and Gibbs gave Clausius and Thomson the credit for creating and developing thermodynamics. There is evidence that Rankine was not even thought of highly within the engineering community [85]. Likely he spread himself too thin.

Hutchinson writes of Rankine that “I shall conclude that apart from his taking the extremely important step of introducing the entropy function, Rankine’s contributions to theoretical thermodynamics as it finally emerged were minimal.” “Even with the entropy function, it was left to Clausius to discover the law of entropy increase, a law which Rankine argued against”. The “somewhat negative conclusions I reach and the positive conclusion about the entropy function are of considerable importance in the historiography of thermodynamics, where Rankine’s theoretical thermodynamics has often been misrepresented” [83].

The same objection to his thermodynamic function (superficially equivalent to Clausius’ entropy) can be made to his “metabatic” and “metabaric” functions; all three of these, as defined, are not true state functions [86]. Rankine writes that Carnot’s function (Thomson’s μ) varies as the inverse of the metabatic function; he also writes that the metabatic function is the absolute temperature. Along with these seemingly meaningful sentences are phrases like “The function of actual energy, efforts, and passive accidents ...” and “If the absolute temperature of any uniformly hot substance be divided into any number of equal parts, the effects of those parts in causing work to be performed are equal.” Maxwell sardonically noted difficulty with this latter phrase [87].

This author would prefer to credit Rankine only for obtaining a mathematical form that gave the appearance of entropy.

Nevertheless, Rankine often had impressive physical intuition, and knew where thermodynamics was needed in developing the steam engine. It is perhaps not coincidental that some five years after publication of his work on the steam engine, steam engines improved significantly in efficiency. Much of Rankine’s work used a questionable vortex model of the atom, but one that was sufficiently close to the truth that it served him as a useful heuristic device. Moreover, Rankine, as a prolific writer of engineering textbooks, surely did not have the time for rigor, and perhaps rigor was not part of his mind-set. The word over-committed comes to mind.

Rankine’s works are available online [88]. In Rankine’s favor are the names “potential energy” and “adiabatic”, as well as the revival of Young’s “energy”. Also in his favor is his recognition that at low absolute temperature the isotherms and adiabats converge [89]. Moreover, he invented the Rankine cycle to describe steam engines [90].

For a work with a higher opinion of Rankine’s contributions, see Ref. [91]. Gibbs, with the first American doctorate in engineering, certainly was not critical of Rankine: “Rankine was attacking the problem in his own way, with one of those marvelous creations of the imagination of which it is so difficult to estimate the precise value.” Moreover, Gibbs wrote that “It may be added that entropy as defined by Clausius is synonymous with the thermodynamic function as defined by Rankine” [92]. However, it remains unclear to the present author that Rankine had a full and uncluttered appreciation of his incomplete “function” (which lacked an integration constant function depending on temperature but not on volume [86]). Moreover, when applied to non-equilibrium situations, Rankine believed his ‘thermodynamic function’ to, in time, decrease rather than increase. I do not share Gibbs’s opinions of Rankine’s work. On the other hand, I note the more sympathetic Daub [93,94].

My own, semi-informed, view of Rankine is a bit like Churchill’s famous commentary on Russia: “It is a riddle, wrapped in a mystery, inside an enigma.” Those who have worked on, or will work on, disentangling the diverse thermodynamic contributions of Rankine deserve commendation.

## 17. On Entropy Production and Wasted Energy

The theory of irreversible processes in bulk, as in Thomson’s studies of heat flow, had to wait until Onsager’s work of 1931 [95]. Callen’s postulational approach to thermodynamics came close to treating irreversible thermodynamics across surfaces [96].

By the time loss of “mechanical effect” by juxtaposition of two materials with surface temperature difference ΔT was considered [97,98,99], the physics community had lost sight of it [100]. One result obtained thereby, and not surprisingly, is that, just as the volume rate of entropy production varies as ${\left(\vec{\nabla }T\right)}^{2}$ [95], so the surface rate of entropy production varies as ${\left(\Delta T\right)}^{2}$ [99].

## 18. Conclusions

This work traces the origins of thermodynamics, with an emphasis on the logical order of the concepts. We note that the material-independent efficiency of a reversible heat engine defines only a well-ordered set of numbers corresponding to temperature, consistent with Thomson’s proposal of two distinct material-independent temperature scales, the second being the ideal gas scale in Kelvin units. We further note that, with the ideal gas temperature $T_{g}$, Clausius’s analysis leads to $\oint dQ/T_{f}=0$ for all paths in *p*-*v* space, and thus to the entropy function $dS=dQ/T_{f}$. This then defines the thermodynamic temperature ${T=\partial U/\partial S|}_{v}$, which (up to a temperature-scale-determining constant) satisfies $T=T_{g}$.

We conclude that, despite Thomson’s great efforts to establish a reliable theoretical basis for temperature, it was not until 1854, when Clausius defined the equivalent of entropy, that a unique thermodynamic temperature could be defined. Despite its abstractness, it appears that entropy is more fundamental than the more accessible temperature. Another way to see the primary nature of entropy is from Boltzmann’s statistical definition $S=k_{B}\ln\Omega$, where Ω is the number of states available to the system, for a given average energy *U*.
